# HAO-AVP: An Entropy-Gini Reinforcement Learning Assisted Hierarchical Void Repair Protocol for Underwater Wireless Sensor Networks

**DOI:** 10.3390/s26020684

**Published:** 2026-01-20

**Authors:** Lijun Hao, Chunbo Ma, Jun Ao

**Affiliations:** School of Information and Communication Engineering, Guilin University of Electronic Technology, Guilin 541004, China

**Keywords:** underwater wireless sensor networks, routing void, hybrid acoustic-optical communication, reinforcement learning, Gini coefficient

## Abstract

Wireless Sensor Networks (WSNs) are pivotal for data acquisition, yet reliability is severely constrained by routing voids induced by sparsity, uneven energy, and high dynamicity. To address these challenges, the Hybrid Acoustic-Optical Adaptive Void-handling Protocol (HAO-AVP) is proposed to satisfy the requirements for highly reliable communication in complex underwater environments. First, targeting uneven energy, a reinforcement learning mechanism utilizing Gini coefficient and entropy is adopted. By optimizing energy distribution, voids are proactively avoided. Second, to address routing interruptions caused by the high dynamicity of topology, a collaborative mechanism for active prediction and real-time identification is constructed. Specifically, this mechanism integrates a Markov chain energy prediction model with on-demand hop discovery technology. Through this integration, precise anticipation and rapid localization of potential void risks are achieved. Finally, to recover damaged links at the minimum cost, a four-level progressive recovery strategy, comprising intra-medium adjustment, cross-medium hopping, path backtracking, and Autonomous Underwater Vehicle (AUV)-assisted recovery, is designed. This strategy is capable of adaptively selecting recovery measures based on the severity of the void. Simulation results demonstrate that, compared with existing mainstream protocols, the void identification rate of the proposed protocol is improved by approximately 7.6%, 8.4%, 13.8%, 19.5%, and 25.3%, respectively, and the void recovery rate is increased by approximately 4.3%, 9.6%, 12.0%, 18.4%, and 24.2%, respectively. In particular, enhanced robustness and a prolonged network life cycle are exhibited in sparse and dynamic networks.

## 1. Introduction

As a key technology for large-scale information acquisition, Wireless Sensor Networks (WSNs) possess immense potential in fields such as resource exploration and security monitoring [1,2,3]. However, in practical applications, characteristics such as node sparsity and dynamic topological changes frequently induce the severe problem of Routing Voids [4]. This phenomenon occurs when a node fails to locate a next-hop relay, leading to link interruption and the formation of communication “dead zones.” This not only causes data loss and a drastic increase in latency but also triggers routing holes or even network paralysis [5,6]. It is noteworthy that although this issue has been studied in traditional static networks, due to fundamental differences in communication media and mobility patterns, existing schemes based on static topologies cannot be directly applied to highly dynamic and complex environments [7,8,9]. Furthermore, most current research fails to adequately address the reliability requirements of heterogeneous networks under resource-constrained conditions [10,11]. Therefore, the design of a specialized and efficient routing void handling mechanism for such complex dynamic networks is urgently required [12].

Specifically, as a critical extension of WSNs into the marine domain, Underwater Wireless Sensor Networks (UWSNs) have garnered significant attention, yet their harsh communication environment renders the void problem even more critical [13]. Unlike terrestrial environments, the underwater channel is characterized by high latency, severe attenuation, and continuous node mobility driven by ocean currents [14]. These unique constraints, coupled with the limited energy of underwater nodes that are difficult to replace, make the network topology highly dynamic and unstable [15]. Consequently, UWSNs face more severe challenges in connectivity and void handling compared to terrestrial networks [16]. In recent years, substantial progress has been achieved in research concerning UWSN routing voids. Early research on pure acoustic networks was mostly based on improved vector forwarding mechanisms. Shi and Zhang et al. [17] proposed the Energy-Aware Vector-Based Forwarding (EA-VBF) protocol, in which the next-hop selection was optimized by introducing a residual energy factor, effectively balancing node energy consumption and relay counts. Subsequently, Saleh et al. [18] proposed an Enhanced Vector-Based Forwarding (VBF) protocol, where a void avoidance mechanism was integrated to bypass communication blind spots by adjusting the routing vector. Although network lifetime has been extended and packet loss reduced to a certain extent by these methods, they were designed primarily for relatively ideal static or low-speed networks. The challenges of dynamic voids in complex underwater environments were not fully considered, leading to performance degradation when nodes are sparse or topology changes frequently.

To overcome the limitations of basic vector forwarding in complex topologies, various avoidance strategies based on density perception and intelligent optimization have been proposed. Ullah et al. [19] proposed a routing scheme based on node density, utilizing local density information to dynamically adjust forwarding strategies, thereby improving the survival rate of data packets in sparse regions. Yang et al. [20] applied a greedy discrete Particle Swarm Optimization (PSO) algorithm for cluster-based routing planning, avoiding potential routing traps through global optimization. Similarly, Mahdi et al. [21] proposed a traffic-aware routing protocol (PG-RES) based on pressure gradient and resistance functions. By comprehensively evaluating node depth, load, and residual energy to dynamically adjust transmission paths, this method effectively alleviates network hotspot issues and ensures the reliability of data forwarding. Meanwhile, Liu et al. [22] pointed out the issue of propagation errors in underwater mobile target localization, indirectly corroborating the difficulty of precise location perception. Despite the avoidance effects being improved by the aforementioned methods through intelligent algorithms, high overhead and latency are incurred by complex calculations and frequent signaling interactions when facing drastically changing topologies, and the success rate of avoidance is difficult to guarantee, leading to avoidance failures. However, preventative avoidance is not always sufficient; when avoidance fails, effective repair techniques become the key to ensuring communication. Khan et al. [23] designed an energy-efficient clustering protocol based on arithmetic progression, in which link robustness was enhanced by optimizing cluster head selection. Ahmad et al. [24] proposed a cooperative routing protocol, while Ye et al. [25] designed a routing strategy based on a multi-dimensional trust model and a void-avoidance algorithm. By, respectively, leveraging node cooperation mechanisms and the capability to identify malicious and void nodes, these methods significantly enhance the efficiency of path repairing and the security of data forwarding. Furthermore, regarding severe network segmentation, the utilization of AUVs for auxiliary repair has become a research hotspot. Wang et al. [26] and Chu et al. [27] explored load balancing based on Q-learning and adaptive reward shaping mechanisms, respectively, providing intelligent support for AUV path planning. The effectiveness of utilizing AUVs as mobile relays or for path repair was further demonstrated by Zhu et al. [28], who proposed the Two-Step Adaptive Path Repair (T-SAPR) protocol, and by the research of Kaiser et al. [29,30,31]. However, these methods mostly belong to passive triggering or rely on expensive external resources; consequently, high scheduling costs and slow response speeds are incurred, making it difficult to adequately cope with the rigorous requirements for real-time performance in high-dynamic environments.

With the increasing demand for network intelligence and high bandwidth, the research paradigm has gradually shifted towards the integration of Reinforcement Learning (RL) technology and hybrid acoustic-optical architectures. RL technology has been widely introduced to endow networks with intelligent decision-making capabilities [32]. He et al. [33] proposed a trust update mechanism based on RL, enhancing the security adaptability of the network. Gao et al. [34] designed a Q-learning load balancing protocol that effectively prolonged the network life cycle. Wang et al. [35] and Nandyala et al. [36] proposed depth-information-based RL opportunistic routing and the Q-learning based Trust Aware Routing (QTAR) protocol, respectively, where multi-dimensional state information was further integrated to realize adaptive perception of dynamic topologies [37]. Although the intelligence level of networks has been significantly improved by these RL protocols, most focus on the optimization of conventional routing metrics, and there is still room for exploration in active void prediction and avoidance. Moreover, in the field of hybrid acoustic-optical networks, relevant research is still in its infancy. Zhu et al. [38] proposed the Priority-based Hybrid Void Prediction (PHVP) protocol, which is considered a representative work in this field, attempting to solve the void problem through packet grading and acoustic-optical collaborative processing. Although the potential of cross-medium transmission was preliminarily demonstrated, a prediction mechanism for void formation is lacking in this method, and the repair means are relatively single-modal, failing to fully leverage the intelligent collaborative advantages of hybrid networks [39].

Despite multi-perspective explorations in existing research, three severe challenges are still faced when applied to complex hybrid acoustic-optical networks: (1) Existing routing protocols are limited to local energy sensing when solving the routing void problem, lacking macro control over overall network load balancing. Metrics from economics that measure fairness are rarely introduced into routing decisions by traditional schemes, making it difficult to effectively quantify the equilibrium and fairness of network energy distribution. Consequently, the network is forced to passively endure the formation of routing void rather than actively avoiding them from the source. (2) The applicability of prediction and identification mechanisms for routing voids in dynamic underwater environments remains to be improved. Existing methods are mostly oriented towards static or low-speed pure acoustic networks. They often rely on periodic interaction or complex calculations, which limits their application in hybrid networks. Furthermore, passive identification mechanisms lead to significant latency and overhead. Currently, a collaborative mechanism fusing energy trend prediction with lightweight real-time detection is lacking. This deficiency makes it difficult to precisely anticipate and lock onto voids in the early stages of their formation. (3) Existing void repair strategies are relatively single-modal in their approaches, making it difficult to balance repair costs and success rates. A one-size-fits-all approach, such as single-medium detouring or premature invocation of expensive auxiliary resources, is adopted by current protocols. In hybrid acoustic-optical networks, further in-depth research is required on how to synergistically utilize the high speed of optical communication and the long distance of acoustic communication to construct a graded progressive repair system ranging from low-cost adjustment to high-cost intervention.

To address the aforementioned challenges, the HAO-AVP protocol is proposed in this paper. The core of this protocol lies in constructing a comprehensive scheme that integrates active avoidance, intelligent decision-making, and graded repair. At the routing decision level, a Reinforcement Learning (RL) framework is introduced. Within this framework, Information Entropy and the Gini Coefficient are incorporated into the reward function. This design actively avoids routing voids from the source by quantifying and optimizing the equilibrium of energy distribution and the fairness of load allocation. Meanwhile, at the void handling level, a collaborative mechanism for prediction, identification, and repair is established. A Markov chain energy prediction model is fused with on-demand hop discovery identification technology. Furthermore, a four-level progressive strategy is designed, comprising intra-medium adjustment (optical path adjustment), cross-medium hopping (acoustic-optical switching), path backtracking, and AUV assisted repair. This ensures that high-cost means are enabled strictly level-by-level only after low-cost schemes prove ineffective, thereby achieving efficient resource utilization. The main research contributions of this paper are as follows:

1. An intelligent routing decision algorithm based on reinforcement learning is proposed. Subsequently, the Gini Coefficient and Information Entropy are introduced into the reward function to quantify the fairness of network energy allocation, which serves to guide load balancing, actively avoid void formation from the source, and prolong the network life cycle.
2. Addressing the challenges of difficult underwater void prediction and single-modal repair means, a prediction-identification-repair collaborative mechanism is proposed. By fusing Markov prediction with on-demand hop discovery technology, the effects of anticipating node failure risks and locking onto void regions are achieved.
3. A four-level progressive repair strategy synergizing acoustic and optical advantages is proposed. Means such as intra-medium adjustment and cross-medium hopping are adaptively matched based on the principle of minimum cost, achieving the unified effect of low overhead, high delivery rate, and strong robustness.

## 2. Problem Statement

In this work, an intuitive description and a formal definition of the “Routing Void” problem within the underwater environment are first presented. The fundamental differences between the characteristics of underwater and terrestrial networks are detailed to clarify the realistic scenarios and technical challenges addressed in this study. Subsequently, the network topology, communication, and energy consumption models are established.

**Figure 1:**
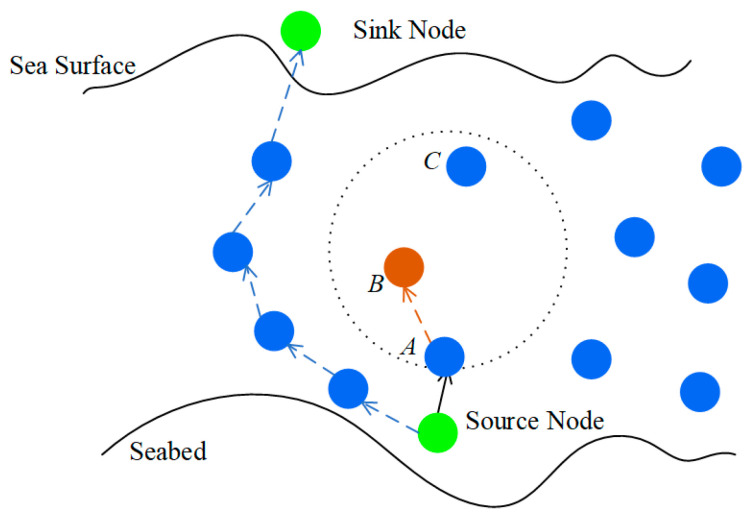
Schematic Diagram of Routing Hole.

In geographic-based underwater greedy routing strategies, neighbors that are closer to the Sink node than the forwarding node itself are invariably preferred as the next hop. However, due to the sparsity of underwater node deployment and complex submarine terrain obstacles, this forwarding strategy is prone to falling into a “local optimum” dilemma, known as a routing void. A typical routing void scenario is illustrated in Figure 1.

It is assumed that a data packet is emitted by a source node and successfully transmitted to Node B via relay Node A. At this juncture, a search for a next-hop relay node is initiated by Node B. However, within the communication range of Node B (indicated by the dashed circle), only a single neighbor, Node C, is located. Unfortunately, the Euclidean distance from Node C to the Sink node is significantly greater than that from Node B to the Sink (i.e., Node C is situated behind Node B). According to greedy forwarding rules, no next-hop node capable of providing positive geographic progress can be found by Node B. Consequently, the data transmission link is interrupted at this point, and Node B is identified as a void node. In the absence of an effective recovery mechanism, the data packet will be discarded, and subsequent data flows directed towards Node B will be trapped in a dead loop while consuming valuable energy, thereby severely compromising network performance.

It is noteworthy that although the routing void problem is also encountered in Terrestrial Wireless Sensor Networks (TWSNs), the direct application of terrestrial solutions (such as the Right-Hand Rule based on planar graph traversal) is rendered ineffective in underwater environments. This is attributed to the unique physical characteristics and network architecture inherent to the underwater environment. As illustrated in Table 1, a detailed comparison between TWSNs and UWSNs is conducted across multiple dimensions, including communication media, propagation characteristics, topological structures, and the causes of voids.

As indicated in Table 1, the fundamental disparities between the two are primarily manifested in the following aspects: Regarding communication media and latency discrepancies, TWSNs rely on Radio Frequency (RF) waves, characterized by an extremely high propagation speed (≈3 × 10^8^ m/s), rendering link latency negligible. In contrast, underwater acoustic communication is predominantly adopted by UWSNs, where the propagation speed of acoustic waves in water is extremely low (≈1.5 × 10^3^ m/s), resulting in excessively high propagation delays. Consequently, void detection methods based on frequent handshakes or real-time topology sensing, which are utilized on land, would incur unacceptable temporal overheads in the underwater environment. In terms of spatial dimensionality, TWSNs are typically modeled as two-dimensional planar networks with relatively static nodes. Conversely, UWSNs are typical three-dimensional networks that exhibit high dynamicity due to the influence of ocean currents. This renders void recovery algorithms based on two-dimensional geometry (such as triangulation) difficult to directly extend to the three-dimensional dynamic space. Regarding void characteristics, voids in terrestrial networks are mostly caused by static obstacles or initial deployment, possessing relatively fixed morphologies. However, voids in underwater networks originate not only from sparse deployment but are also more frequently generated dynamically due to node energy depletion or movement with water currents. This phenomenon of “dynamic voids” necessitates stronger adaptability and predictive capabilities in handling mechanisms. In summary, traditional void handling strategies designed for terrestrial environments are unable to adapt to underwater scenarios characterized by high latency, high dynamicity, and three-dimensional features. Therefore, the design of a specialized routing void prediction and recovery mechanism for underwater hybrid acoustic-optical networks is deemed particularly urgent.

## 3. Underwater Hybrid Acoustic-Optical Network Model Building

The list of symbols is presented in Table 2, in which all key notations utilized in the equations and algorithms are defined to facilitate quick reference.

### 3.1. Network Topology Model

In this paper, the UWSN is modeled as an undirected *graph* G=(V,E) within a three-dimensional Euclidean space. All physical entities within the network are represented by the vertex set $V=\{v_{1},v_{2},\ldots ,v_{N}\}\cup \{v_{s}\}$, where the i-th ordinary sensor node is denoted by $v_{i}$, and its coordinate in the three-dimensional space is given by $\left(x_{i},y_{i},z_{i}\right)$, where $z_{i}$ represents the deployment depth of the node. The unique Sink Node, situated at the center of the water surface, is represented by $v_{s}$, which is responsible for collecting all underwater data and communicating with the onshore data center. All potential communication links between nodes are represented by the edge set E. An edge $(v_{i},v_{j})\in E$ is considered to exist if and only if the distance between node $v_{i}$ and node $v_{j}$ is less than or equal to the maximum communication range of a specific communication mode. In the hybrid acoustic-optical network, the edge set E can be further decomposed into the union of the acoustic communication link set $E_{a}$ and the optical communication link set $E_{o}$, which is expressed as $E=E_{a}\cup E_{o}$.

An acoustic communication link $(v_{i},v_{j})\in E_{a}$ is considered to exist provided that the condition $d(v_{i},v_{j})\leq R_{a}$ is satisfied, where $R_{a}$ denotes the maximum effective range of underwater acoustic communication. An optical communication link $(v_{i},v_{j})\in E_{o}$ is established if and only if two conditions are simultaneously met:

1. Distance Condition: The condition $d(v_{i},v_{j})\leq R_{o}$ must be satisfied, where $R_{o}$ represents the maximum effective range of underwater optical communication.
2. Angle Condition: Node $v_{j}$ must be located within the coverage range of the beam divergence angle of node $v_{i}$, and vice versa.

In this topology model, $R_{o}$ is utilized as the upper distance threshold for defining the existence of potential links, while the specific issue of angular alignment is further considered in the routing decision process. Typically, it is observed that $R_{o}\ll R_{a}$.

**Figure 2:**
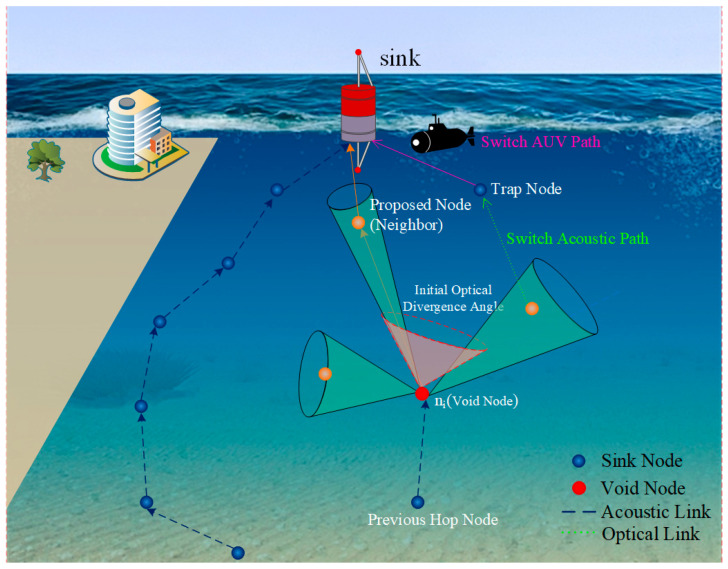
Network Model.

A neighbor node set $N(v_{i})=\{v_{j}|(v_{i},v_{j})\in E\}$ is maintained by each node $v_{i}$, which can be similarly subdivided into an acoustic neighbor set $N_{a}(v_{i})$ and an optical neighbor set $N_{o}(v_{i})$. The network model is illustrated in Figure 2.

Sensor node $n_{i}$ is equipped with independent acoustic and optical transceivers, possessing dual-mode communication capabilities. The energy of nodes is constrained, with the initial energy denoted as $E_{init}$. It is assumed that the three-dimensional coordinates $\left(x_{i},y_{i},z_{i}\right)$ of each node can be acquired via a specific localization algorithm, and the residual energy $E_{res}$ can be sensed. Node positions are subject to dynamic changes due to the impact of ocean currents. The Sink Node is situated at the water surface and is unrestricted in energy; it is responsible for collecting data uploaded from all underwater nodes and communicating with the onshore data center via RF links. The AUV serves as a mobile auxiliary unit, equipped with acoustic-optical communication capabilities and rechargeable energy sources. Within this protocol, beyond routine cruising missions, the AUV is dispatchable for the execution of routing repair tasks.

### 3.2. Communication Model

1. Underwater Acoustic Communication Model

The energy consumption associated with underwater acoustic communication is primarily influenced by the propagation loss of signals within the aqueous medium. According to the Thorp model [40,41], the path loss $TL_{a}(d_{ij},f)$ (unit: dB) from node $v_{i}$ to node $v_{j}$ can be calculated via the following formula:

(1) $$TL_{a}(d_{ij},f)=\tilde{k}\cdot 10\log_{10}(d_{ij})+d_{ij}\cdot a(f)$$

where $d_{ij}$ represents the distance between the transmitting and receiving nodes (in meters); f denotes the frequency of the acoustic wave (in kHz); $\tilde{k}$ is the spreading factor, which is typically set to 1.5 (corresponding to practical spreading) or 2 (corresponding to spherical propagation); and a(f) denotes the frequency-dependent absorption coefficient, the empirical formula of which is given by:

(2) $$10\log_{10}a(f)=\frac{0.11f^{2}}{1+f^{2}}+\frac{44f^{2}}{4100+f^{2}}+2.75\times 10^{-4}f^{2}+0.003$$

The Signal-to-Noise Ratio (SNR) at the receiving node can be expressed as:

(3) $$SNR_{a}=SL-TL_{a}(d_{ij},f)-NL+DI$$

where SL represents the Source Level, NL denotes the Ambient Noise Level, and DI stands for the Directivity Index. The power spectral density of ambient noise, N(f), is typically synthesized from four primary noise sources: turbulence noise $N_{t}(f)$, shipping noise $N_{s}(f)$, wave noise $N_{w}(f)$, and thermal noise $N_{th}(f)$. According to empirical formulas [4], these noise components (in dB) can be expressed as follows:

(4) $$10\log N_{t}(f)=17-30\log f$$

(5) $$10\log N_{s}(f)=40+20(s-0.5)+26\log f-60\log(f+0.03)$$

(6) $$10\log N_{w}(f)=50+7.5\sqrt{w}+20\log f-40\log(f+0.4)$$

(7) $$10\log N_{th}(f)=-15+20\log f$$

where s denotes the shipping activity factor (ranging from 0 to 1), and w represents the wind speed at the sea surface (in m/s). The total power spectral density of ambient noise is expressed as the sum of these four components:

(8) $$N(f)=N_{t}(f)+N_{s}(f)+N_{w}(f)+N_{th}(f)$$

The transmission power $P_{tx\_a}$ required for transmitting a data packet of length L (bits), and the power $P_{rx\_a}$ consumed by the node for receiving said packet, are, respectively, given by:

(9) $$P_{tx\_a}\propto 10^{(TL_{a}(d_{ij},f)+NL-DI+SNR_{th})/10}$$

(10) $$P_{rx\_a}=P_{elec}$$

where $SNR_{th}$ represents the minimum SNR threshold required for successful demodulation at the receiver, and $P_{elec}$ denotes the fixed power consumption for circuit processing.

2. Underwater Optical Communication Model

The path loss of underwater optical communication is primarily induced by absorption and scattering, following Beer-Lambert’s law. The optical power $P_{rx\_opt}$ received at the receiver can be expressed as:

(11) $$P_{rx\_opt}(d_{ij})=P_{tx\_opt}\cdot \eta_{t}\eta_{r}\cdot \frac{A_{rx}}{A_{beam}(d_{ij})}\cdot e^{-c(\lambda )d_{ij}}$$

where $P_{tx\_opt}$ denotes the optical transmission power. $\eta_{t}$ and $\eta_{r}$ represent the optical efficiencies of the transmitter and receiver, respectively. The aperture area of the receiver is denoted by $A_{rx}$. The area of the optical beam at a distance $d_{ij}$ is indicated by $A_{beam}(d_{ij})$, which is approximated as $\pi {\left(d_{ij}\tan(\theta /2)\right)}^{2}$, where θ represents the beam divergence angle. Furthermore, c(λ) denotes the beam attenuation coefficient, which is dependent on the optical wavelength λ and the water quality.

Shot noise and thermal noise are identified as the primary sources of noise in communication. Consequently, the SNR at the receiver, denoted as $SNR_{opt}$, is expressed as:

(12) $$SNR_{opt}=\frac{{\left(R\cdot P_{tx\_opt}\right)}^{2}}{\sigma_{shot}^{2}+\sigma_{thermal}^{2}}$$

where $P_{tx\_opt}$ represents the optical transmission power; R denotes the responsivity of the photodetector, with a typical value of approximately 0.5 A/W; and $\sigma_{shot}^{2}$ and $\sigma_{thermal}^{2}$ represent the variances of shot noise and thermal noise, respectively, which can be specifically expressed as:

(13) $$\sigma_{shot}^{2}=2qB(R\cdot P_{rx\_opt}+I_{bg}A_{rx}R+I_{d})$$

(14) $$\sigma_{thermal}^{2}=\frac{4k_{B}T}{R_{L}}B$$

where *q* represents the elementary charge, with a value of $1.602\times 10^{-19}$ Coulombs; $P_{rx\_opt}$ denotes the received optical power, which is calculated via the path loss formula. $I_{bg}$ signifies the background light irradiance, which is influenced by factors such as sunlight and bioluminescence, exhibiting a wide variation range from $10^{-5}\;{W/m}^{2}$ (in the deep sea) to $10^{2}\;{W/m}^{2}$ (near the water surface). $A_{rx}$ is the receiver aperture area, determined by the dimensions of the receiving lens; B represents the bandwidth, which is associated with the target communication rate; $k_{B}$ is the Boltzmann constant, with a value of $1.38\times 10^{-23}$ J/K; T denotes the absolute temperature, which can be set to 283 K; and $R_{L}$ is the load resistance. Furthermore, $I_{d}$ represents the detector dark current, which is dependent on the material and quality of the detector.

3. Energy Consumption Model

The energy consumption incurred by node $v_{i}$ in transmitting a data packet of length L (bits) to node $v_{j}$, denoted as $E_{tx}(L,d_{ij})$, and the energy consumption for receiving said data packet, denoted as $E_{rx}(L)$, are categorized into two modes: acoustic and optical.

Acoustic Mode:

(15) $$E_{tx\_a}(L,d_{ij})=P_{tx\_a}(d_{ij})\cdot (L/R_{a})$$

(16) $$E_{rx\_a}(L)=P_{rx\_a}\cdot (L/R_{a})$$

where $R_{a}$ denotes the data rate of acoustic communication.

Optical Mode:

(17) $$E_{tx\_o}(L,d_{ij})=P_{tx\_opt}(d_{ij})\cdot (L/R_{o})$$

(18) $$E_{rx\_o}(L)=P_{rx\_opt}\cdot (L/R_{o})$$

where $R_{o}$ denotes the data rate of optical communication. The initial energy of each node $v_{i}$ is denoted as $E_{init}$, and its residual energy at time t is represented by $E_{rem}(v_{i},t)$.

In UWSNs, the objective of routing is to identify a reliable and efficient path from the source node $v_{src}$ to the sink node $v_{s}$. In greedy routing strategies based on geographic location or depth, a node $v_{j}$ that offers the maximum “progress” among the neighbors is selected by node $v_{i}$ as the next hop. Progress is typically defined as the difference between the distance from the current node to the destination and the distance from the next-hop node to the destination.

Routing Void Node: A node $v_{i}$ currently forwarding a data packet is designated as a Routing Void Node if, within its entire set of neighbor nodes $N(v_{i})$, there exists no node $v_{j}$ such that $v_{j}$ is closer to the destination sink node than $v_{i}$. This can be described mathematically as:

(19) $$\forall v_{j}\in N(v_{i}),\;d(v_{j},v_{s})\geq d(v_{i},v_{s})$$

In addition to the inherent sparsity of the network, the formation of routing voids is significantly attributed to the premature energy depletion of certain nodes (such as nodes in “hotspot” regions near the Sink Node), which is caused by the assumption of excessive data forwarding tasks. Consequently, a “dead zone” composed of failed nodes is formed within the network topology, which similarly results in routing interruptions.

In summary, based on the underwater hybrid acoustic-optical network system model constructed above, it is observed that significant heterogeneity, dynamicity, and energy constraints are exhibited by the network environment. Constrained by the strict “Line-of-Sight” (LoS) transmission requirements of underwater optical communication and the inherent high propagation latency of acoustic communication, coupled with the continuous positional drift of nodes induced by ocean currents, endogenous structural contradictions are faced by the system during actual operation. Specifically, data packets are extremely prone to being trapped in “Routing Voids” with no available path during the greedy forwarding process, due to the sparsity of node deployment and unpredictable topological changes. Simultaneously, given that underwater node batteries are difficult to replace, the premature failure of critical relay nodes is caused by the uneven distribution of network loads due to rapid energy consumption; this, in turn, induces “Routing Voids” or even leads to large-scale network paralysis. The efficient operation of the network is rendered difficult to maintain by traditional single-medium routing or simple greedy forwarding strategies, owing to the communication dead zones and energy efficiency bottlenecks derived from the inherent characteristics of the model. Therefore, to overcome the aforementioned model limitations, the design of a data transmission mechanism characterized by high reliability, low latency, and energy balance within a dynamic environment is deemed necessary.

## 4. Proposed HAO-AVP Protocol

**Figure 3:**
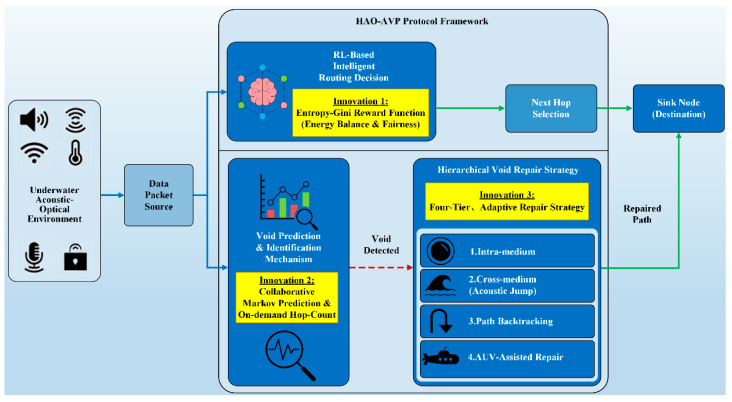
Overall Architecture of the HAO-AVP Protocol.

In consideration of the severe challenges wherein routing voids and communication interruptions are prone to be induced by limited node energy and dynamic topological changes in underwater hybrid acoustic-optical networks, the HAO-AVP is proposed in this paper. The overall architecture of the HAO-AVP protocol is illustrated in Figure 3. Two core mechanisms are systematically integrated into this framework: First, an intelligent routing decision-making mechanism based on reinforcement learning is adopted, which aims to actively avoid voids through load balancing. Second, a “prediction-identification-repair” collaborative void handling mechanism is constructed, which is utilized to passively cope with existing or impending routing interruptions.

### 4.1. Reinforcement Learning-Based Routing Decision-Making via Entropy and Gini Coefficient

To address the issues of high dynamicity in underwater network topology and uneven resource allocation, the Q-Learning algorithm within RL is introduced in this paper, whereby nodes are endowed with intelligent next-hop decision-making capabilities. Simultaneously, it is taken into consideration that the reward functions of traditional reinforcement learning algorithms are confined to local communication gains, and a macroscopic grasp of the global network load status is lacking. Consequently, an improved reinforcement learning routing strategy is proposed, in which Information Entropy and the Gini Coefficient are integrated into the design of the reward function. Through this approach, the optimization of local transmission efficiency is achieved while network-wide load balancing and fairness are simultaneously taken into account.

#### 4.1.1. States, Actions, and Q-Value Functions

In this paper, the routing decision process is modeled as a Markov Decision Process (MDP).

State (s): For any node $v_{i}$ holding a data packet, its state $s_{i}$ is defined as a vector containing critical local information that influences the decision-making process.

(20) $$s_{i}=[E_{rem}(v_{i}),D(v_{i}),Q_{len}(v_{i})]$$

where $E_{rem}(v_{i})$ denotes the normalized residual energy of node $v_{i}$; $D(v_{i})$ represents the distance from node $v_{i}$ to the sink node $v_{s}$; and $Q_{len}(v_{i})$ indicates the packet queue length of node $v_{i}$.

Action (a): The action space $A(s_{i})$ of node $v_{i}$ is defined as the set of all its neighbor nodes $N(v_{i})$. The execution of an action $a_{j}\in A(s_{i})$ corresponds to the selection of neighbor node $v_{j}$ as the next hop for forwarding the data packet.

Q-Value Function: The Q-value function $Q(s_{i},a_{j})$ represents the long-term expected reward obtainable by selecting node $v_{j}$ as the next hop under state $s_{i}$. The update of the Q-value is governed by the classic Bellman equation:

(21) $$Q_{t+1}(s_{i},a_{j})=(1-\alpha )\cdot Q_{t}(s_{i},a_{j})+\alpha [R(s_{i},a_{j})+\gamma \underset{a^{\prime }\in A(s_{j})}{\max}Q_{t}(s_{j},a^{\prime })]\;$$

where α∈[0,1] represents the learning rate; $R(s_{i},a_{j})$ denotes the immediate reward obtained subsequent to the execution of action $a_{j}$; γ∈[0,1] is the discount factor; and $\underset{a^{\prime }\in A(s_{j})}{\max}Q_{t}(s_{j},a^{\prime })$ signifies the maximum potential future Q-value obtainable in the subsequent state $s_{j}$.

#### 4.1.2. Reward Function Design Based on Entropy and Gini Coefficient

The reward function $R(s_{i},a_{j})$ is constituted by three components:

(22) $$R(s_{i},a_{j})=w_{1}\cdot R_{prog}(v_{j})+w_{2}\cdot R_{energy}(v_{j})+w_{3}\cdot R_{balance}(v_{i},v_{j})\;$$

where $w_{1}$, $w_{2}$, and $w_{3}$ are defined as the weighting coefficients, where the condition $w_{1}+w_{2}+w_{3}=1$ is satisfied.

1. Progress Reward $R_{prog}(v_{j})$:
  (23) $$R_{prog}(v_{j})=\frac{d(v_{i},v_{s})-d(v_{j},v_{s})}{\underset{v_{k}\in N(v_{i})}{\max}\{d(v_{i},v_{s})-d(v_{k},v_{s})\}}\;$$
2. Energy Reward $R_{energy}(v_{j})$:
  (24) $$R_{energy}(v_{j})=E_{rem}(v_{j})$$
3. Equilibrium Reward $R_{balance}(v_{i},v_{j})$:

This reward is calculated within the neighbor node set $N(v_{i})$ of node $v_{i}$; it is jointly determined by the energy information entropy and the forwarding load Gini coefficient, and is regulated by a dynamic weighting factor $\omega_{b}$.

(25) $$R_{balance}(v_{i},v_{j})=\omega_{b}(v_{i})\cdot \left(1-G_{load}(v_{i})\right)+(1-\omega_{b}(v_{i}))\cdot \frac{H_{energy}(v_{i})}{\log_{2}(|N(v_{i})|)}$$

Dynamic Weighting Factor $\omega_{b}(v_{i})$: The emphasis placed on fairness (the Gini Coefficient) and equilibrium (Entropy) is dynamically adjusted by this factor, in accordance with the standard deviation $\sigma_{E}$ of the energy distribution among neighbor nodes.

(26) $$\omega_{b}(v_{i})=\tanh\left(k_{1}\cdot \sigma_{E}(v_{i})\right)$$

where $k_{1}$ is defined as the adjustment parameter, and $\sigma_{E}(v_{i})=\sqrt{\frac{1}{\left|N(v_{i})\right|}\sum_{v_{k}\in N(v_{i})}{\left(E_{rem}(v_{k})-{\bar{E}}_{rem}\right)}^{2}}$ denotes the standard deviation of the residual energy of neighbor nodes. When a significant energy discrepancy is observed, $\omega_{b}$ approaches 1, whereby greater emphasis is placed on load fairness; conversely, the equilibrium of the overall energy distribution is prioritized.

Energy Information Entropy $H_{energy}(v_{i})$: The energy proportion $p_{k}$ is expressed as $p_{k}=E_{rem}(v_{k})/\sum_{v_{m}\in N(v_{i})}E_{rem}(v_{m})$.

(27) $$H_{energy}(v_{i})=-\sum_{v_{k}\in N(v_{i})}p_{k}\log_{2}(p_{k})$$

Forwarding Load Gini Coefficient ($G_{load}(v_{i})$): It is assumed that the number of data packets forwarded by each neighbor node $v_{k}$ within the past time window is denoted as $L_{k}$.

(28) $$G_{load}(v_{i})=\frac{\sum_{k=1}^{\left|\left.N(v_{i})\right|\right.}\sum_{m=1}^{\left|\left.N(v_{i})\right|\right.}\left|\left.L_{k}-L_{m}\right|\right.}{2\left|{\left.N(v_{i})\right|}^{2}\bar{L}(v_{i})\right.}$$

where the average load of the neighbor nodes is represented by $\bar{L}(v_{i})=\frac{1}{\left|N(v_{i})\right|}\sum_{k=1}^{\left|\left.N(v_{i})\right|\right.}L_{k}$.

4. Design Principles of the Compound Reward Function and Theoretical Implications on Convergence Behavior

To ensure the convergence and robustness of the routing strategy within the highly dynamic underwater environment, a compound reward function comprising three complementary terms is proposed in this paper. Each term addresses a specific optimization objective, collectively reshaping the Q-value manifold within the Markov Decision Process (MDP), thereby influencing the convergence behavior of the agent:

Composition and Theoretical Role of Reward Terms

(1) Progress Reward $R_{prog}$: Providing Directional Gradient.

This term serves as the fundamental driving force for routing convergence. By rewarding nodes capable of providing positive geographic progress toward the sink node, a gradient field with explicit directionality is constructed within the Q-value space by $R_{prog}$. Theoretically, random walks or loops are prevented by this mechanism, ensuring that the selected path strictly converges geometrically toward the destination.

(2) Energy Reward $R_{energy}$: Establishing Feasibility Constraints.

Although the shortest path is guaranteed by $R_{prog}$, the survivability of nodes is neglected. The selection of nodes with low residual energy is penalized by $R_{energy}$, introducing an energy-aware constraint. During the convergence process, the attractors of the Q-value landscape are corrected by this term, shifting the convergence point of the optimal policy from the geometric shortest path to an energy-sustainable path, thereby preventing premature link breakage caused by single-point depletion.

(3) Balance Reward ($R_{balance}$): Enhancing Robustness via Regularization.

This constitutes the core innovation of the proposed protocol. By integrating Information Entropy and the Gini coefficient, $R_{balance}$ functions as a regularization term, imposing a penalty on behaviors where traffic is concentrated on a single optimal node. The peaks of the globally optimal solution in the Q-value function are smoothed, encouraging a probabilistic distribution of traffic among multiple healthy neighbors.

Impact on Convergence Behavior under Dynamic Topology

Under conditions of highly dynamic topology changes, winner-takes-all convergence is often induced by traditional greedy strategies. This state of convergence is fragile—once the optimal node fails due to movement or energy depletion, severe oscillations are faced by the Q-value table. Upon the introduction of the Entropy-Gini term $R_{balance}$, the convergence objective is transformed from a static single optimal solution to a dynamic multi-path routing set. This probabilistic convergence behavior enables the agent to maintain multiple high-value neighbors simultaneously. When drastic changes occur in the network topology, natural redundancy is provided by this wide policy space, allowing the algorithm to rapidly switch to backup paths, thereby maintaining the stability of the convergence state.

#### 4.1.3. Learning and Decision-Making Process

A Q-table is maintained by each node $v_{i}$. When a data packet is available for transmission, the next hop $v_{j}^{*}$ is selected by adopting the ε-greedy strategy.

(29) $$v_{j}^{*}=\left\{\begin{array}{cc} \arg\underset{v_{j}\in N(v_{i})}{\max}Q(s_{i},a_{j}) & \mathrm{if}\;\tilde{q}>\varepsilon \\ \mathrm{random}\;\mathrm{choice}\;\mathrm{from}\;N(v_{i}) & \mathrm{if}\;\tilde{q}\leq \varepsilon \end{array}\right.$$

where $\tilde{q}$ represents a random number distributed within the interval [0,1]. Upon the successful forwarding of a data packet, information regarding the next state is acquired by node $v_{i}$ via mechanism such as listening or ACK. Subsequently, its Q-table is updated in accordance with the Bellman equation (Equation (21)).

In summary, the specific execution flow of the reinforcement learning-based intelligent next-hop decision-making process is summarized in Algorithm 1. It is detailed in this algorithm how the local state is observed by a node, and how the Q-value is iteratively updated through the calculation of a composite reward function incorporating energy entropy and the Gini coefficient, whereby intelligent routing decision-making is realized.
**Algorithm 1:** RL-Based Routing with Entropy & Gini RewardInput: Current Node $n_{i}$
$,\;\mathrm{Neighbor}\;\mathrm{Set}\;N_{i}$
Output: Next Hop $n_{next}$
1:  $\mathrm{Observe}\;\mathrm{State}\;S_{t}=\left(E_{res},Dist_{\sin k},Q_{len}\right)$ //2: // Action Selection (Epsilon-Greedy)3: 
IF random()<ε
$\;\mathrm{THEN}\;\mathrm{Select}\;\mathrm{random}\;n_{next}$
$\;\mathrm{form}\;N_{i}$
4: 
$\mathrm{ELSE}\;n_{next}=argmax_{n_{j}}Q\left(S_{t},n_{j}\right)$
5: 
$\mathrm{Forward}\;\mathrm{Packet}\;\mathrm{to}\;n_{next}$
$\;\mathrm{and}\;\mathrm{Observe}\;S_{t+1}$
6: // Reward Calculation (Core Innovation)7:  $\mathrm{Compute}\;R_{progress}$$\;\mathrm{and}\;R_{energy}$ using Equations (23) and (24)8: 
$\mathrm{Compute}\;R_{equilibrium}$ based on Entropy Equation (27) & Gini Equation (28)9: 
$R_{total}=w1\cdot R_{progress}+w2\cdot R_{energy}+w3\cdot R_{equilibrium}$
10: // Update Q-Value11: 
$Q(S_{t},n_{next})=(1-\alpha )\cdot Q(S_{t},n_{next})+\alpha \cdot (R_{total}+\gamma \cdot max(Q(S_{t+1})))$


### 4.2. Collaborative Routing Void Handling Mechanism

In response to the issue wherein the formation of underwater routing voids is characterized by concealment and difficulty in real-time capture, a Markov chain model is introduced to actively predict the energy depletion trends of nodes. Simultaneously, given that the risk of false alarms exists in a single probability prediction model and sudden interruptions in the physical topology cannot be perceived, a collaborative void prediction and identification mechanism is proposed. Within this mechanism, background energy trend prediction is deeply integrated with foreground on-demand hop discovery technology, whereby the timely perception of potential circuit break risks by nodes is ensured, and void regions are precisely locked.

#### 4.2.1. Markov Chain-Based Void Prediction

Potential routing voids are predicted by monitoring the residual energy variation trends of neighbor nodes. The energy state of each neighbor node $v_{j}$ is divided into M discrete levels, denoted as $S=\{S_{1},S_{2},\ldots ,S_{M}\}$. An M×M energy state transition probability matrix, $P_{j}$, is constructed by observing the frequency of transitions between different energy levels over a long period.

Using this Markov model, the probability $\pi_{j}^{\left(k\right)}(S_{crit})$ that node $v_{j}$ transitions to the “critical” state $S_{crit}$ after k time synchronization steps, given the current state $S_{x}$, can be predicted as follows:

(30) $$\pi_{j}^{\left(k\right)}=\pi_{j}^{\left(0\right)}\cdot {\left(P_{j}\right)}^{k}$$

where $\pi_{j}^{\left(0\right)}$ is defined as the initial state probability distribution vector. A routing void warning is triggered when the joint probability $P_{void}$, representing the scenario where the entire set of “progress” neighbors of node $v_{i}$, denoted as $N_{prog}(v_{i})=\{v_{j}\in N(v_{i})|d(v_{j},v_{s})<d(v_{i},v_{s})\}$, enters the “critical” state within the next k steps—exceeds the warning threshold $P_{th}$.

(31) $$P_{void}=\prod_{v_{j}\in N_{prog}(v_{i})}\pi_{j}^{\left(k\right)}(S_{crit})>P_{th}$$

#### 4.2.2. Void Identification via On-Demand Hop Discovery

A lightweight, on-demand hop discovery mechanism is initiated when node $v_{i}$ is confirmed to be trapped in a routing void (i.e., $N_{prog}(v_{i})=Ø$). A “VOID_DISCOVERY” request packet containing a Time-To-Live (TTL) limit is broadcast by $v_{i}$ to all its neighbors. Upon receipt of this request, the hop count to the sink node, $H(v_{j},v_{s})$, is returned by neighbor $v_{j}$, provided that $v_{j}$ is not a void node itself. By collecting this feedback, whether an accessible path exists in the vicinity can be rapidly ascertained by $v_{i}$, whereby a basis for selecting a recovery strategy is provided.

In summary, to achieve precise anticipation and rapid localization of voids, the detailed execution steps of the collaborative void prediction and identification algorithm are summarized in Algorithm 2. Through the coordination of background trend prediction and foreground real-time detection, the timely perception of potential circuit break risks by nodes is ensured by this algorithm.
**Algorithm 2:** Void Prediction and IdentificationInput: Neighbors $N_{i}$
$,\;\mathrm{Energy}\;\mathrm{History}\;H_{E}$
1: // --- Proactive Prediction (Background Process) ---2:
$\mathrm{Update}\;\mathrm{Markov}\;\mathrm{Transition}\;\mathrm{Matrix}\;\mathrm{based}\;\mathrm{on}\;H_{E}$
3:
$\mathrm{Calculate}\;\mathrm{Probability}\;P_{danger}$ of neighbors entering “Endangered State”4:
$\mathrm{IF}\;Joint_{Probability}(P_{danger})>Threshold_{warning}$ THEN5: Trigger Void Alert and update Neighbor List6: END IF7: // --- Real-time Identification (Forwarding Process) ---8: IF no neighbor provides progress to Sink THEN9:
$\mathrm{Broadcast}\;VOID_{DISCOVERY}$(TTL)10:
$\mathrm{Receive}\;Hop_{Count}$ feedback from neighbors11: IF valid path exists THEN Update Routing Table12: ELSE Trigger Hierarchical Repair (Algorithm 3)13: END IF

### 4.3. Graded Void Repair Mechanism

To address the issues of link interruption and packet loss caused by underwater routing voids, conventional path backtracking or single-medium repair mechanisms are introduced to attempt communication recovery. Simultaneously, it is considered that balancing repair success rate and network energy consumption is difficult for existing single repair means in complex hybrid acoustic-optical environments (e.g., limited coverage of purely optical repair and excessive energy consumption of purely acoustic repair). Therefore, a graded progressive void repair strategy is proposed, wherein intra-medium optical path adjustment, cross-medium acoustic hopping, path backtracking, and AUV-assisted repair are deeply integrated. The dual optimization of high delivery rate and low energy consumption is realized, while the lowest cost solution is adaptively matched according to the severity of the void. Upon confirmation of a routing void, a four-level progressive repair strategy is initiated by the HAO-AVP protocol.

1. Level 1: Intra-medium Adjustment (Optical Path Adjustment)

If the data packet is currently being transmitted via optical communication and a void is encountered, repair is attempted by node $v_{i}$ within the optical communication medium. The objective of this strategy is to identify a minimum beam divergence angle increment Δθ. Let $\theta_{old}$ be denoted as the initial divergence angle. The set of uncovered progress neighbors is defined as:

(32) $$N_{prog}^{\prime }=\{v_{j}\in N_{o}(v_{i})\backslash N_{covered}|d(v_{j},v_{s})<d(v_{i},v_{s})\}$$

The minimum required new divergence angle is given by:

(33) $$\theta_{new}=\underset{v_{j}\in N_{prog}^{\prime }}{\min}\{2\phi_{ij}\}+\delta_{margin}$$

where $\phi_{ij}$ denotes the deviation angle of $v_{j}$, and $\delta_{margin}$ represents the angular margin. The energy cost associated with increasing the divergence angle can be modeled as:

(34) $$C_{1}(\theta_{new})=E_{tx\_o}\cdot {\left(\frac{\tan(\theta_{new}/2)}{\tan(\theta_{old}/2)}\right)}^{2}$$

Decision Rule: This repair is executed provided that $N_{prog}^{\prime }\neq Ø$, $\theta_{new}\leq \theta_{max}$, and $C_{1}(\theta_{new})<T_{1}$ (cost threshold). Otherwise, the process proceeds to the second level.

2. Level 2: Cross-medium Hopping (Acoustic-Optical Switching)

If optical path adjustment fails, “cross-medium hopping” is performed by utilizing the long-distance characteristics of acoustic communication. A Comprehensive Detour Utility Evaluation Function (CDUE), denoted as $U(v_{j})$, is designed in this protocol to guide the decision-making process:

(35) $$U(v_{j})=w_{q}\cdot \left(\frac{FP(v_{j})}{\underset{v_{k}\in N_{a}(v_{i})}{\max}FP(v_{k})+\epsilon }\cdot e^{\frac{E_{rem\_avg}(v_{j})}{E_{init}}}\right)+w_{r}\cdot \mathrm{PAPR}(d(v_{i},v_{j}),f)-w_{c}\cdot \frac{E_{tx\_a}(L,d(v_{i},v_{j}))}{E_{rem}(v_{i})}$$

where $w_{q}$, $w_{r}$, $w_{c}$ are defined as weighting coefficients, and ϵ is a small constant used to prevent division by zero. $E_{rem\_avg}(v_{j})$ denotes the average residual energy of the optical neighbors of $v_{j}$. $FP(v_{j})$ represents the forward optical potential of node $v_{j}$:

(36) $$FP(v_{j})=\left|\left\{v_{k}\in N_{o}(v_{j})|d(v_{k},v_{s})<d(v_{j},v_{s})\right\}\right|$$

PAPR(d,f) is defined as the predicted packet reception rate of the acoustic link:

(37) $$\mathrm{PAPR}(d,f)={\left(1-P_{b}(d,f)\right)}^{L}$$

where $P_{b}(d,f)$ denotes the Bit Error Rate (BER), and L represents the packet length.

Decision Rule: The acoustic neighbor $v_{j}^{*}$ that maximizes the comprehensive detour utility is selected by node $v_{i}$ as the next hop:

(38) $$v_{j}^{*}=\arg\underset{v_{j}\in N_{a}(v_{i})}{\max}U(v_{j})$$

The cost of this operation is defined as the energy consumption of acoustic communication, $C_{2}=E_{tx\_a}(L,d(v_{i},v_{j}^{*}))$.

3. Level 3: Path Backtracking

Path backtracking is initiated when node $v_{i}$ is trapped in a complete void. Upon receipt of the $\mathrm{BACKTRACK}(v_{i},H)$ message, a new next hop $v_{k}^{*}$ must be intelligently selected by the previous hop node $v_{p}$ to avoid falling into the void again. To this end, a Backtrack Utility (BU) function is designed:

(39) $$v_{k}^{*}=\arg\underset{v_{k}\in A^{\prime }(s_{p})}{\max}\{Q(s_{p},a_{k})\cdot LS{\left(v_{p},v_{k}\right)}^{\eta }-\lambda_{1}\cdot \exp\left(-\frac{d{\left(v_{k},v_{i}\right)}^{2}}{2\sigma^{2}}\right)\}$$

where $A^{\prime }(s_{p})=N(v_{p})\backslash ({\mathrm{Blacklist}}_{p}\cup H)$ is defined as the new action space.

Model Derivation and Analysis: The concept of risk aversion in backtracking scenarios is embodied by this decision model. $Q(s_{p},a_{k})$ is maintained as the basis for decision-making, representing the long-term expected return of selecting node $v_{k}$. $LS{\left(v_{p},v_{k}\right)}^{\eta }$ is defined as the link stability factor, where $LS(v_{p},v_{k})\in [0,1]$ denotes the link quality evaluated based on historical communication success rates, and η≥1 represents the risk aversion coefficient. As η increases, a stronger inclination is shown by the decision-making process toward selecting links with extremely stable historical performance.

The final term is identified as a Gaussian penalty term. A penalty is imposed on candidate nodes $v_{k}$ that are geographically proximate to the known void node $v_{i}$. Where $d(v_{k},v_{i})$ denotes the distance between the two nodes, σ controls the range of the penalty, and $\lambda_{1}$ represents the intensity of the penalty. The introduction of this penalty term is based on a reasonable assumption: voids are typically regional in nature; therefore, a higher risk of becoming trapped in a void is possessed by nodes located near a known void point. Through this penalty term, data packets are guided to actively bypass the explored void regions.

If $A^{\prime }(s_{p})=Ø$, node $v_{p}$ is considered to be trapped in a void as well, and backtracking is continued to its previous hop.

4. Level 4: AUV-Assisted Repair

When backtracking is repeatedly observed in a region, indicating the existence of large-scale network partitioning, AUV-assisted repair is triggered.

Dynamic Joint Evaluation Model of Task Urgency and Repair Benefit: A comprehensive priority score $S_{k}$ is evaluated by the AUV for each repair request k (originating from node $v_{k}$):

(40) $$\begin{array}{c} S_{k}=\left(w_{sev}\cdot \log\left(1+Count_{bt}\left(k\right)\right)+w_{aff}\cdot \left|N_{aff}(k)\right|\right)\cdot \left(1+w_{qlen}\cdot {\bar{Q}}_{len}\left(k\right)\right)\\ -\left(w_{dist}\cdot \frac{d\left(P_{AUV},Loc_{k}\right)}{D_{max}}+w_{energy}\cdot \frac{E_{travel}\left(k\right)}{E_{AUV\_rem}}+w_{dev}\cdot \mathrm{Dev}\left(k\right)\right) \end{array}$$

where the w series are defined as weighting coefficients. $Count_{bt}(k)$ is denoted as the backtrack counter, $\left|N_{aff}\left(k\right)\right|$ represents the number of affected neighbors, ${\bar{Q}}_{len}\left(k\right)$ is the regional average queue length, $d\left(P_{AUV},Loc_{k}\right)$ is the navigation distance, $E_{travel}\left(k\right)$ is the travel energy consumption, and $\mathrm{Dev}\left(k\right)$ denotes the degree of deviation from the main mission route.

Calculation of Optimal Repair Point (PSO): Upon determination of the task, the optimal repair position $P^{*}$ is solved by the AUV via the PSO algorithm. The fitness function is given by:

(41) $$F\left(P\right)=w_{c1}\cdot M_{conn}\left(P\right)-w_{d}\cdot d\left(P_{AUV},P\right)$$

where $M_{conn}\left(P\right)$ represents the quantity of void-surrounding nodes that can be connected at position P. Particle Velocity Update:

(42) $$\Theta_{m}(t+1)=\tilde{\omega }\cdot \Theta_{m}(t)+c_{1}r_{1}(P_{best,m}-P_{m}(t))+c_{2}r_{2}(G_{best}-P_{m}(t))$$

Particle Position Update:

(43) $$P_{m}(t+1)=P_{m}(t)+\Theta_{m}(t+1)$$

where ω is the inertia weight, $c_{1}$ and $c_{2}$ are learning factors, $r_{1}$ and $r_{2}$ are random numbers, $P_{best,m}$ denotes the historical best position of particle m, and $G_{best}$ represents the global best position.

Repair Mode Decision:

Temporary Relay Cost:

(44) $$C_{relay}=C_{travel}(P^{*})+C_{op}\cdot T_{stay}$$

New Node Deployment Cost:

(45) $$C_{deploy}=C_{travel}(P^{*})+C_{node}$$

Decision Rule:

(46) $$\mathrm{Action}=\left\{\begin{array}{ll} \mathrm{Deploy}\;\mathrm{Node} & \mathrm{if}\;C_{deploy}<C_{relay}\\ \mathrm{Act}\;\mathrm{as}\;\mathrm{Relay} & \;\mathrm{otherwise} \end{array}\right.$$

Through this set of graded repair mechanisms, the most appropriate solution is flexibly and efficiently selected by the HAO-AVP protocol according to the severity of the void. Valuable network resources are conserved to the maximum extent while network reliability is ensured.

In summary, to minimize network energy consumption while guaranteeing the repair success rate, this cost-aware four-level progressive repair logic is summarized as Algorithm 3. The principle of “low cost priority” is followed by this algorithm, wherein intra-medium adjustment, cross-medium hopping, path backtracking, and AUV assistance are sequentially attempted through logical judgment until a new forwarding path is successfully established.
**Algorithm 3:** Hierarchical Void Repair StrategyInput: Void Node $n_{void}$, Data Packet P1: // Level 1: Intra-medium Adjustment (Optical)2:
IF (Medium=Optical$)\;\mathrm{AND}\;(Cost(New_{Angle})<Threshold$) THEN3:   Adjust Divergence Angle and Retransmit P; RETURN Success4: END IF 5: // Level 2: Cross-medium Jumping (Acoustic)6: Calculate CDUE for all acoustic neighbors using Equation (35)7:
IF max(CDUE)>0 THEN8:   Switch to Acoustic Mode; Forward P to best neighbor; RETURN Success9: END IF10: // Level 3: Path Backtracking11:
$\mathrm{Send}\;Backtrack_{Msg}$
$\;\mathrm{to}\;Previous_{Hop}$
12:
$Previous_{Hop}$ selects new path using Backtrack Utility Equation (39)13:
$\mathrm{IF}\;Path_{Found}$ THEN RETURN Success14: // Level 4: AUV-Assisted Repair15: Request AUV assistance16: AUV calculates Optimal Position using PSO Equations (41)–(43)17: AUV performs Relay or Deployment based on Cost Equation (46); RETURN Success

## 5. Simulation Experiments and Performance Analysis

The performance of the proposed HAO-AVP is comprehensively evaluated in this chapter through simulation experiments. HAO-AVP is compared with five representative existing protocols, RLORP-DI, PHVP, ERR-UWSN, SOVHAR, and T-SAPR, under various network scenarios. Furthermore, the advantages of the proposed protocol in terms of reliability, efficiency, and load balancing are verified via a series of performance metrics.

### 5.1. Simulation Environment and Parameter Settings

1. Simulation Environment

The simulation experiments in this study are implemented on the MATLAB R2013b platform. A three-dimensional underwater network simulation environment is constructed to simulate the node deployment, mobility, communication, and energy consumption of hybrid acoustic-optical networks. The communication and energy consumption models described in Section 4 are integrated into this environment, whereby key processes such as packet generation, multi-hop forwarding, and void formation and repair can be simulated. The specific hardware configurations and software environment parameters adopted in the experiments are presented in Table 3.

2. Simulation Parameters

In order to align the simulation environment more closely with realistic underwater scenarios and to ensure the comparability of experimental results, classic literature in relevant fields was primarily referenced for the simulation parameters in this study. The detailed parameter settings are presented in Table 4.

3. Performance Evaluation Metrics

To comprehensively evaluate protocol performance, the following five core metrics are adopted in this paper. All numerical values of the indicators are obtained by averaging the results of multiple simulation runs to eliminate errors caused by randomness.

Packet Delivery Ratio (PDR): This is defined as the ratio of the total number of data packets successfully reaching the sink node to the total number of data packets sent by all source nodes. A higher PDR indicates that stronger reliability and void handling capability are possessed by the protocol. Its calculation formula is given by:

(47) $$\mathrm{PDR}=\frac{P_{received}}{P_{sent}}\times 100\%$$

where $P_{received}$ denotes the total number of unique data packets successfully received by the sink node, and *P_sent_* represents the total number of data packets sent by all source nodes in the network.

Average End-to-End Delay: The communication efficiency of the network is reflected by this metric. It is defined as the average time consumed by all successfully delivered data packets from their generation at the source node to their final reception by the sink node. Lower latency indicates that higher efficiency is achieved by the protocol. Its calculation formula is expressed as:

(48) $${\mathrm{Delay}}_{\mathrm{avg}}=\frac{\sum_{k=1}^{P_{received}}\left(T_{arrival,k}-T_{generate,k}\right)}{P_{received}}$$

where $T_{arrival,k}$ represents the time at which the k-th successfully received data packet arrives at the sink node, and $T_{generate,k}$ denotes the time at which the said data packet is generated at the source node.

Network Lifetime: This metric is utilized to measure the durability and overall energy efficiency of the network. In this paper, it is defined as the duration from the commencement of network operation until the first sensor node fails (dies) due to energy depletion. A longer lifetime indicates that stronger energy management and load balancing capabilities are demonstrated by the protocol. Its mathematical definition is given by:

(49) $$\mathrm{Lifetime}=\min\left\{t|\exists v_{j}\in V,E_{rem}\left(v_{j},t\right)\leq 0\right\}$$

where *V* is the set of all nodes in the network, and $E_{rem}\left(v_{j},t\right)$ represents the residual energy of node $v_{j}$ at time t.

Number of Alive Nodes: The overall health status of the network as it evolves over time is dynamically reflected by this metric. It is defined as the quantity of nodes within the network possessing residual energy greater than zero at any given instant t (or at a specific round) during the simulation. Its value at time t is expressed by the following equation:

(50) $$N_{\mathrm{alive}}\left(t\right)=\left|\left\{v_{j}\in V|E_{rem}\left(v_{j},t\right)>0\right\}\right|$$

where |⋅| denotes the cardinality of the set.

Gini Coefficient of Energy Consumption: This metric is employed to quantify the degree of equilibrium in the distribution of residual energy across all nodes in the network, serving as a critical indicator for measuring load balancing performance. The range of the Gini coefficient is [0,1]. A more balanced energy consumption among nodes and a fairer network load allocation are indicated by a value closer to 0, whereas a larger disparity in energy consumption and the existence of severe “hotspot” issues are indicated by a value closer to 1. Its calculation formula is given by:

(51) $$G=\frac{\sum_{i=1}^{N}\sum_{j=1}^{N}\left|E_{rem,i}-E_{rem,j}\right|}{2N\sum_{k=1}^{N}E_{rem,k}}$$

where N represents the total number of nodes in the network, and $E_{rem,i}$ and $E_{rem,j}$ denote the residual energy of node i and node j, respectively.

### 5.2. Simulation Results and Analysis

#### 5.2.1. Performance Analysis with Varying Number of Nodes

**Figure 4:**
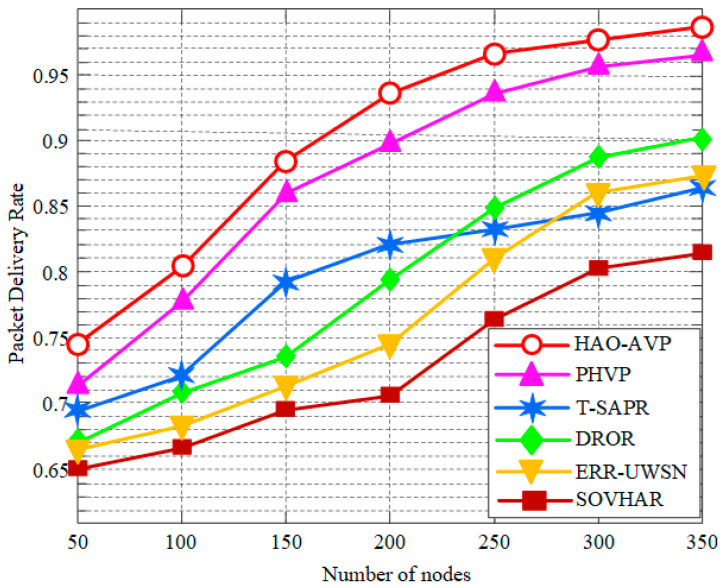
PDR vs. Number of Nodes.

**Figure 5:**
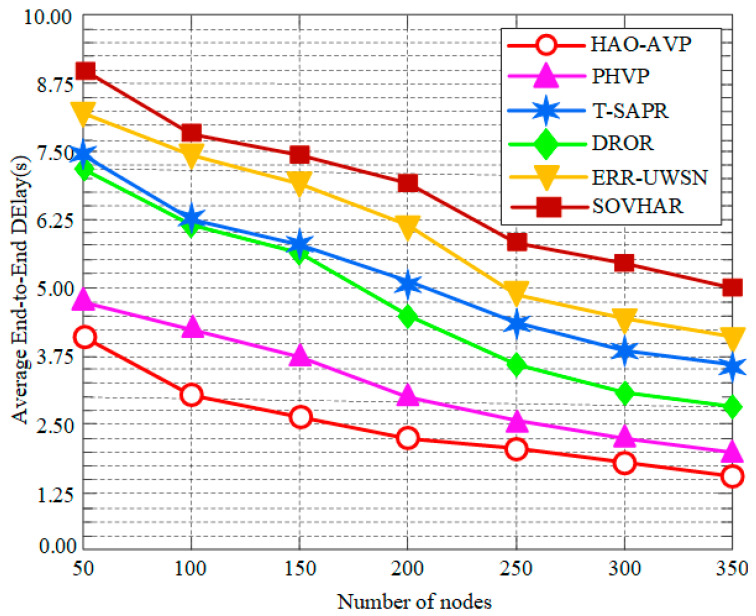
Average End-to-End Delay vs. Number of Nodes.

**Figure 6:**
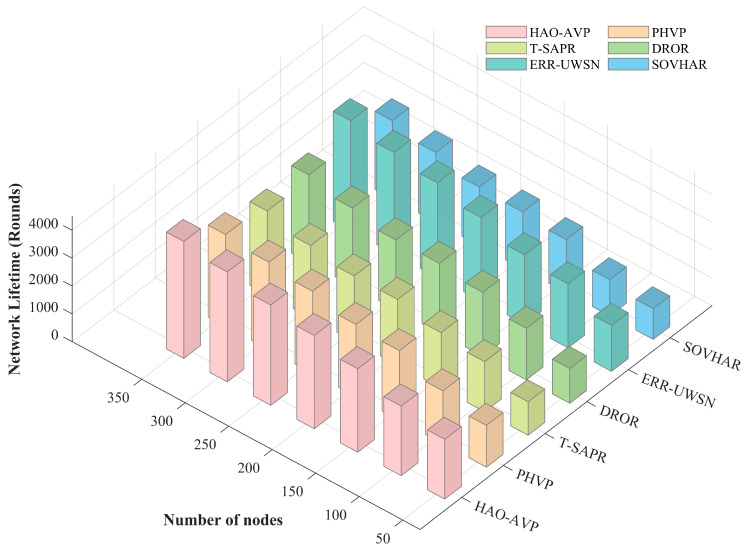
Network Lifetime vs. Number of Nodes.

**Figure 7:**
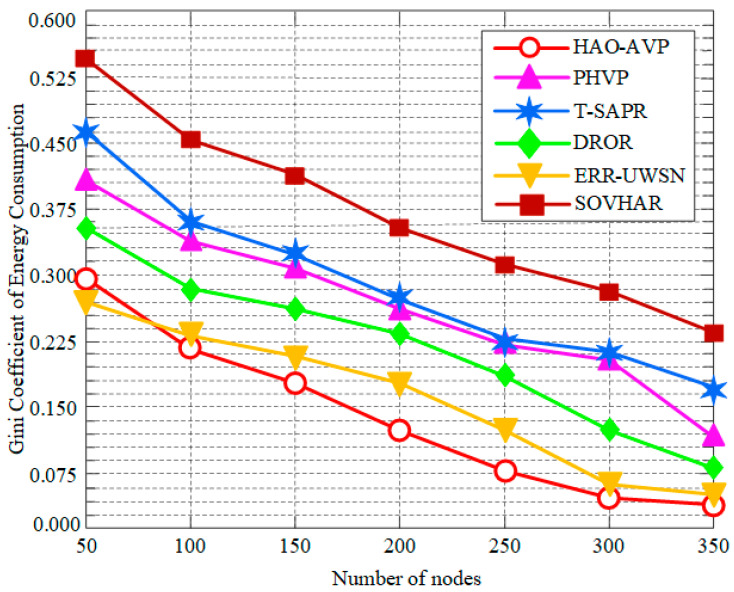
Gini Coefficient of Energy Consumption vs. Number of Nodes.

**Figure 8:**
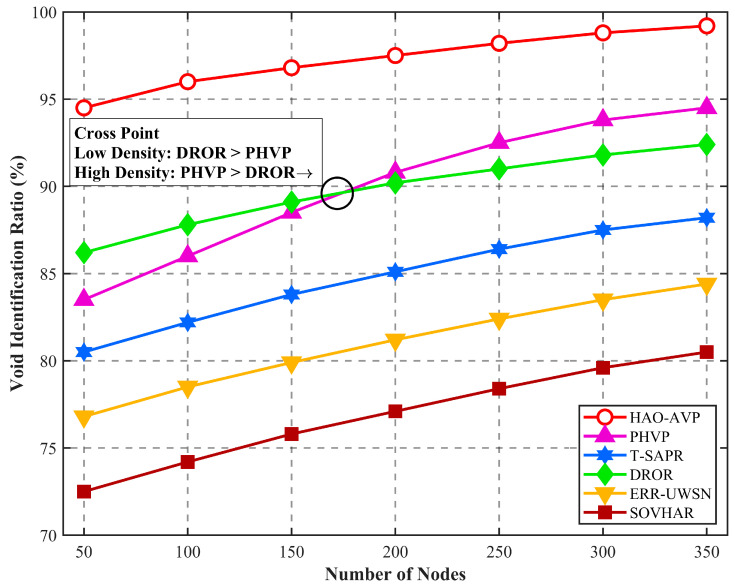
Hole Detection Results vs. Number of Nodes.

**Figure 9:**
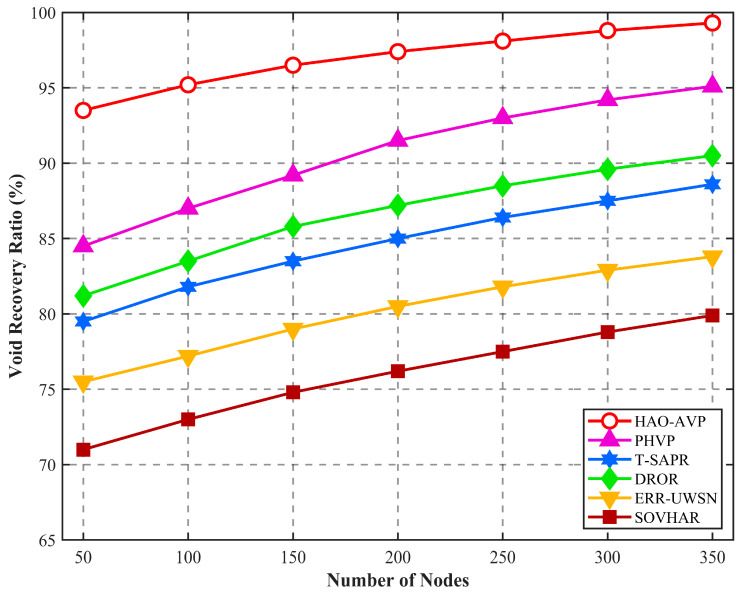
Hole Repair Results vs. Number of Nodes.

In this section, the performance of the proposed HAO-AVP protocol is comprehensively analyzed through simulation experiments under different node densities. Specifically, four core metrics—PDR, Average End-to-End Delay, Network Lifetime, and the Energy Consumption Gini Coefficient—are evaluated. Furthermore, the intrinsic correlations among these metrics are explored. The comparative performance analysis results under varying node densities are illustrated in Figure 4, Figure 5, Figure 6, Figure 7, Figure 8 and Figure 9.

1. Number of Nodes and PDR:

Figure 4 illustrates the relationship between the number of nodes and the PDR. As can be observed from Figure 4, with an increase in the number of nodes, an upward trend in PDR is exhibited by all protocols. This is attributed to the improvement in network connectivity and the increase in path redundancy. By virtue of its advantages in hybrid acoustic-optical communication, RL-based intelligent decision-making, and a robust graded repair mechanism (incorporating “cross-medium hopping” and “AUV assistance”), communication segmentation in sparse networks is effectively bridged by the HAO-AVP protocol. A consistently high PDR is maintained, which approaches saturation as the number of nodes increases, reaching a maximum of approximately 96.8%. At 300 nodes, compared to PHVP, T-SAPR, DROR, ERR-UWSN, and SOVHAR, the PDR of HAO-AVP is improved by 2.08%, 13.54%, 8.33%, 11.45%, and 18.08%, respectively. Limited PDR improvements are shown by other protocols due to limitations in their routing decisions or repair mechanisms. When the density becomes high enough that the network is nearly “fully connected,” the physical upper limit is approached by the PDR, and marginal benefits become negligible.

2. Node Quantity and Average End-to-End Delay:

Figure 5 depicts the relationship between the number of nodes and the average end-to-end delay. As indicated in Figure 5, when the number of nodes is 350, a significant advantage is demonstrated by HAO-AVP. Compared with PHVP, DROR, T-SAPR, ERR-UWSN, and SOVHAR, the delay is reduced by approximately 15.6%, 19.5%, 22.6%, 30.2%, and 33.5%, respectively. This advantage is primarily attributed to the prioritized utilization of high-speed optical links for transmission by HAO-AVP, combined with RL intelligent decision-making to actively avoid congestion, whereby transmission and retransmission times are effectively reduced. In contrast, pure acoustic protocols such as DROR and ERR-UWSN are constrained by the low speed of sound, resulting in high base latency. Although hybrid acoustic-optical communication is utilized by PHVP, a global prediction mechanism is lacking; meanwhile, due to the involvement of waiting times for AUV scheduling in T-SAPR, a larger overall latency is incurred.

3. Node Quantity and Network Lifetime:

Figure 6 illustrates the comparison of the network lifetime under different node densities. As demonstrated in Figure 6, when the number of nodes is 350, a significant advantage is exhibited by the HAO-AVP protocol. Compared with PHVP, T-SAPR, DROR, ERR-UWSN, and SOVHAR, the network lifetime is extended by approximately 27.3%, 32.6%, 35.5%, 7.7%, and 50.0%, respectively. The primary reason for this result is attributed to the fact that Information Entropy and the Gini Coefficient are innovatively incorporated into the Reinforcement Learning reward function by HAO-AVP. Consequently, the quantification and optimization of the fairness of network energy distribution are realized, thereby effectively avoiding the premature failure of critical nodes caused by excessive load (i.e., the “energy hotspot” problem). In contrast, although path connectivity is considered by protocols such as SOVHAR and T-SAPR, a lack of fine-grained control over global load balancing is exhibited, leading to uneven energy consumption among nodes and a shortened overall network lifespan. Furthermore, while an energy-aware mechanism is possessed by ERR-UWSN, its balancing capability in high-density networks is still considered less precise than the entropy-weighted strategy of HAO-AVP.

4. Node Quantity and Energy Consumption Gini Coefficient:

Figure 7 illustrates the relationship between the number of nodes and the energy consumption Gini coefficient. As indicated in Figure 7, a more balanced energy consumption is represented by a lower energy consumption Gini coefficient. The lowest Gini coefficient is maintained by the HAO-AVP protocol across all node densities. This is directly attributed to the optimization of Information Entropy and the Gini Coefficient within its RL reward function, whereby superior load balancing is achieved, and the overuse of hotspots is avoided. For instance, when the number of nodes is 200, the energy consumption Gini coefficient of the proposed method is reduced by 29.8% relative to that of ERR-UWSN. Higher Gini coefficients are exhibited by other protocols due to the lack of explicit guidance for global balancing. In general, as node density increases and path options multiply, a slight decrease in the Gini coefficients is observed across all protocols, which is consistent with the trend of extended network lifetime.

5. Node Quantity and Void Identification Rate and Void Recovery Rate:

Figure 8 illustrates the performance of the void identification rate with respect to the number of nodes. As demonstrated in Figure 8, the performance comparison regarding the void identification rate between the proposed HAO-AVP protocol and five comparative protocols (PHVP, DROR, T-SAPR, ERR-UWSN, and SOVHAR) is presented under different node densities (ranging from 50 to 350 nodes).

Overall, as node density increases, an upward trend in the identification rate is exhibited by all protocols. A consistently high void identification rate is maintained by HAO-AVP, reaching approximately 99.2% when the number of nodes is 350. This is primarily attributed to the integration of the collaborative mechanism involving “Markov trend prediction” and “on-demand hop discovery,” whereby potential circuit breaks are actively perceived, and void boundaries are confirmed in real-time. In contrast, although hierarchical processing is introduced by PHVP, its identification relies mainly on passive feedback, resulting in an identification rate of approximately 94.5%. It is noteworthy that in the low-density region (50–150 nodes), the curve of DROR is slightly higher than that of T-SAPR; however, it is surpassed by T-SAPR as density increases. This is because simple voids can be rapidly discovered in sparse networks by the depth-information-based greedy strategy of DROR, whereas identification bottlenecks are caused by the lack of a global perspective in high-density complex topologies. Conversely, although T-SAPR is initially constrained by the latency of AUV scheduling, as the number of nodes increases, malicious or faulty nodes are more accurately eliminated by its trust-model-based routing decisions, thereby improving the effective identification rate. Relatively limited identification capabilities (84.4% and 80.5%, respectively) are exhibited by ERR-UWSN and SOVHAR when facing large-scale or complex-shaped voids, as they rely primarily on local neighbor information. Richer neighbor information is provided by higher node density. At 350 nodes, compared with PHVP, DROR, T-SAPR, ERR-UWSN, and SOVHAR, the average void identification rate of HAO-AVP is improved by approximately 7.6%, 8.4%, 13.8%, 19.5%, and 25.3%, respectively, validating the effectiveness of the proposed method across different network scales.

Figure 9 illustrates the effect of node quantity on the void recovery rate. As depicted in Figure 9, the trends of void recovery rates for various protocols under different node densities are presented. With an increase in the number of nodes, network connectivity is enhanced, and a steady upward trend in the recovery performance of each protocol is exhibited. Among them, relatively optimal performance is consistently maintained by HAO-AVP. This is attributed to its four-level progressive repair strategy, whereby multiple fault-tolerant defense lines are constructed by flexibly integrating means such as optical adjustment, acoustic hopping, and AUV physical repair. The performance of PHVP ranks second, as single-medium interruptions are effectively alleviated by its acoustic-optical dual-mode switching mechanism. It is noteworthy that the recovery rate of DROR is slightly superior to that of T-SAPR. This is because depth-based multi-path opportunistic forwarding is adopted by DROR, resulting in a wider path search range. In contrast, although AUV assistance is introduced in T-SAPR, nodes that are physically connected but possess low reputation scores may be actively eliminated by its core trust management mechanism; consequently, potential repair paths are sacrificed to a certain extent by this focus on security. Conversely, passive backtracking or local scanning is primarily relied upon by ERR-UWSN and SOVHAR. Consequently, local optima are easily encountered when facing complex dead-end topologies, resulting in limited recovery success rates. In the typical scenario of 350 nodes, compared with PHVP, DROR, T-SAPR, ERR-UWSN, and SOVHAR, the void recovery rate of HAO-AVP is improved by approximately 4.3%, 9.6%, 12.0%, 18.4%, and 24.2%, respectively, fully validating the effectiveness of the multi-modal collaborative repair mechanism.

#### 5.2.2. Performance Analysis Under Different Mobility Speeds

In this section, the robustness of the protocol under varying network dynamics is evaluated. The number of nodes is fixed at 150 (representing medium density). By increasing the maximum mobility speed of nodes from 0 m/s (static) to 6 m/s (highly dynamic), the variations in PDR, Average End-to-End Delay, and Network Lifetime are observed.

**Figure 10:**
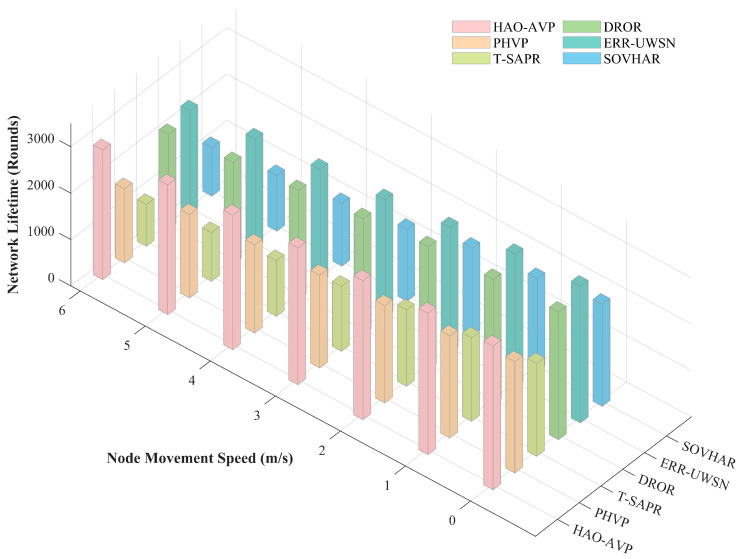
Network Lifetime vs. Node Moving Speed.

1. Node Moving Speed and Network Lifetime:

Figure 10 illustrates the impact of node mobility speed on network lifetime. As indicated by the figure, as the node mobility speed increases from 0 m/s to 6 m/s, the dynamicity of the network topology is significantly enhanced. This leads to frequent link interruptions and routing reconstructions, thereby increasing the energy consumption of nodes; consequently, a downward trend in network lifetime is exhibited by all protocols. However, the longest network lifetime is consistently maintained by the proposed HAO-AVP protocol across all speed settings, demonstrating exceptional robustness. In particular, in the high-dynamic scenario of 6 m/s, the advantage of HAO-AVP is particularly evident. Compared with PHVP, T-SAPR, DROR, ERR-UWSN, and SOVHAR, the network lifetime is extended by approximately 2.1%, 14.1%, 9.2%, 12.6%, and 21.0%, respectively.

The primary reasons for this performance improvement are attributed to the following factors: First, the Gini Coefficient and Information Entropy are introduced into the RL reward function by HAO-AVP. Even under conditions of rapid topological changes, data flows are continuously guided away from low-energy nodes by this mechanism, whereby the equilibrium of energy consumption (Load Balancing) is maintained on a global scale. Second, the risks of circuit breaks are accurately anticipated by the proposed prediction-identification-repair collaborative mechanism, and energy wastage caused by blind retransmissions and invalid path searches is effectively reduced. In contrast, excessive energy consumption is incurred by T-SAPR due to the high computational and signaling overheads brought by its trust evaluation mechanism. Furthermore, the acceleration of node energy exhaustion is caused by the frequent triggering of passive repair strategies in SOVHAR and PHVP under high-dynamic environments.

**Figure 11:**
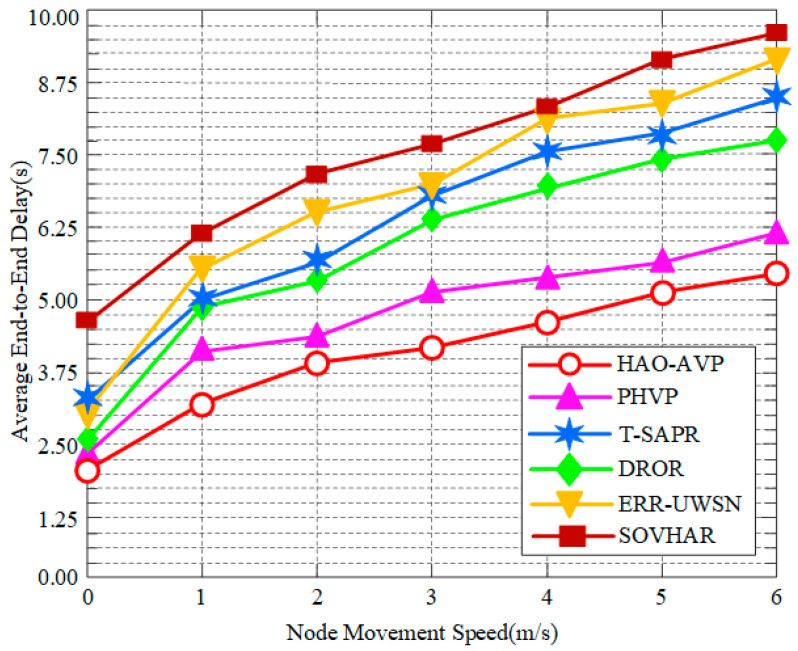
Average End-to-End Delay vs. Node Moving Speed.

2. Node Mobility Speed and Average End-to-End Delay:

Figure 11 illustrates the trends in average end-to-end delay for each protocol under different node mobility speeds. In high-dynamic scenarios with a node mobility speed of 6 m/s, the lowest delay performance is maintained by the HAO-AVP protocol. Compared with PHVP, T-SAPR, DROR, ERR-UWSN, and SOVHAR, the delay is reduced by approximately 11.3%, 21.4%, 27.6%, 30.3%, and 32.5%, respectively. The advantage of HAO-AVP is primarily attributed to the prioritized utilization of high-speed optical communication links and the rapid adaptation to topological changes achieved through RL, whereby routing oscillation and reconstruction time are reduced. In contrast, protocols such as SOVHAR and ERR-UWSN are constrained by the low propagation speed of underwater acoustic channels; furthermore, a drastic increase in latency is caused by the frequent triggering of time-consuming passive repair mechanisms under high-speed mobility. It is noteworthy that a crossover phenomenon is observed between the curves of ERR-UWSN and T-SAPR in the low-speed phase (0–3 m/s). At low speeds, paths are effectively found by the active avoidance strategy of ERR-UWSN, resulting in lower latency than T-SAPR. However, as speed increases, the avoidance mechanism of ERR-UWSN is rendered ineffective by overly rapid topological changes, necessitating frequent retransmissions. Consequently, a rapid rise in latency is exhibited, surpassing that of T-SAPR. Conversely, although high base overhead is incurred by T-SAPR, its sensitivity to dynamicity is relatively lower, resulting in a more moderate increase.

**Figure 12:**
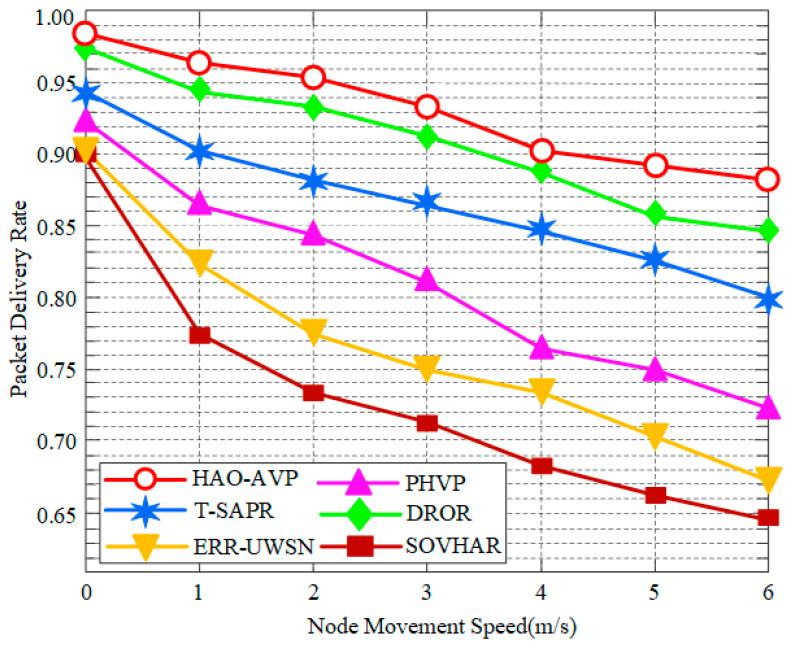
PDR vs. Node Moving Speed.

3. Node Mobility Speed and PDR:

Figure 12 depicts the impact of node mobility speed on PDR. As indicated in the figure, as the node mobility speed increases, changes in network topology become more frequent and drastic, leading to difficulties in link maintenance and unstable neighbor relationships; consequently, a downward trend in PDR is exhibited by all protocols. However, a relatively gentle decline in the PDR curve is shown by the HAO-AVP protocol, demonstrating strong robustness. This is primarily attributed to its core reinforcement learning routing decision mechanism, where continuous learning and adaptation to environmental changes are enabled, allowing for rapid adjustment of routing strategies even during fast topological shifts. Simultaneously, a strong guarantee for maintaining network connectivity in high-dynamic environments is provided by its comprehensive four-level graded repair mechanism, particularly path backtracking and AUV-assisted repair. For instance, when the node mobility speed is 5 m/s, PDR improvements of 18.12%, 7.95%, 4.54%, 23.86%, and 29.54% are achieved by HAO-AVP relative to PHVP, T-SAPR, DROR, ERR-UWSN, and SOVHAR, respectively. Among the comparative algorithms, relatively stable performance is also exhibited by DROR and T-SAPR. Reinforcement learning is similarly utilized by DROR, endowing it with a certain degree of adaptability to partially mitigate the effects of mobility. Although the basic routing of T-SAPR is significantly affected by mobility, physical-layer connection restoration during severe link interruptions is provided by its AUV-assisted repair mechanism, thereby maintaining a relatively high PDR, despite the potential latency associated with this repair. In contrast, more distinct declines in PDR with increasing speed are observed for PHVP, ERR-UWSN, and SOVHAR, due to their relatively slow response to topological changes or limited repair mechanisms. Although PHVP is a hybrid protocol, the adaptability of its routing decisions and repair mechanisms to rapid topological changes is considered inferior to that of RL-based protocols. The active avoidance strategy of ERR-UWSN is liable to fail more frequently when topology changes rapidly. Similarly, large-scale or frequently changing voids caused by rapid node movement are difficult to cope with using the local detouring strategy of SOVHAR.

**Figure 13:**
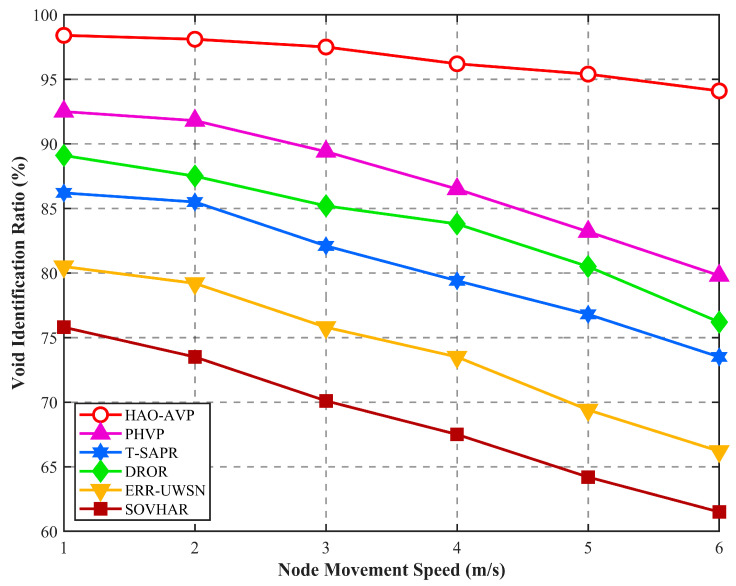
Impact of Node Moving Speed on Hole Detection Results.

4. Node Mobility Speed and Void Identification Rate/Void Recovery Rate:

Figure 13 presents the results regarding the impact of node mobility speed on the void identification rate. As can be seen from the figure, as the node mobility speed increases from 1 m/s to 6 m/s, the dynamicity of the network topology is intensified, leading to a downward trend in the void identification performance of all protocols. However, by virtue of the collaborative Markov prediction mechanism, node failure risks are actively anticipated by HAO-AVP, allowing strong robustness to be maintained in high-speed scenarios. In contrast, PHVP is limited by the maintenance lag of its packet grading structure, resulting in a decline in the identification rate as speed increases. It is noteworthy that the performance of DROR is superior to that of T-SAPR. This is because the depth-based opportunistic forwarding strategy adopted by DROR possesses stronger adaptability to real-time topological changes, whereas reliance is placed on trust model updates by T-SAPR, where the convergence speed of trust values is found to lag behind topological changes under high-speed mobility, leading to the misjudgment of certain nodes. The most significant performance degradation is observed in ERR-UWSN and SOVHAR due to their reliance on local passive sensing, which makes it difficult to keep pace with high-speed topological changes. In the high-dynamic limit scenario of 6 m/s, compared with PHVP, DROR, T-SAPR, ERR-UWSN, and SOVHAR, the void identification rate of HAO-AVP is improved by approximately 17.5%, 23.7%, 28.8%, 42.4%, and 54.1%, respectively, fully validating the effectiveness of the active prediction mechanism in dynamic networks.

**Figure 14:**
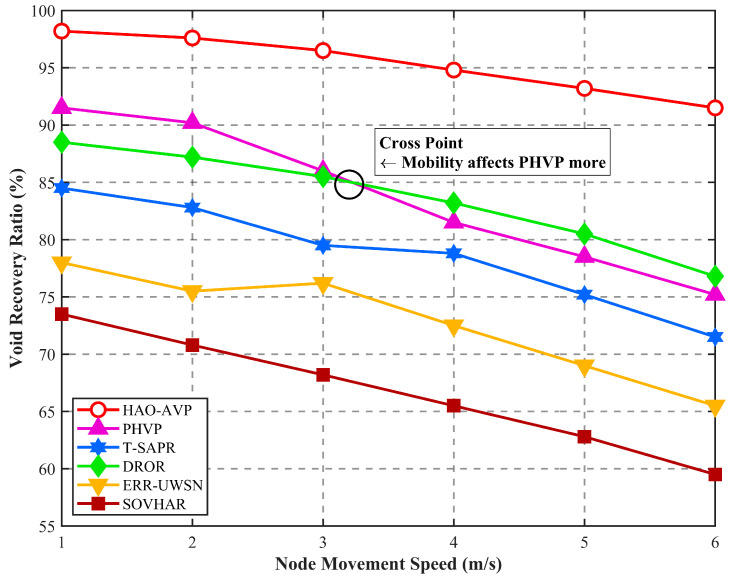
Impact of Node Moving Speed on Hole Repair Results.

Figure 14 illustrates the results regarding the impact of node mobility speed on the void recovery rate. As indicated by the figure, as the node mobility speed increases from 1 m/s to 6 m/s, a downward trend in the void recovery rates of all protocols is induced by high-frequency variations in network topology; however, significant differences in the attenuation magnitudes are observed among the various protocols. The relatively highest recovery performance is maintained by HAO-AVP throughout the entire testing interval. This is attributed to the fact that link ruptures are perceived in advance by its collaborative prediction mechanism, and rapid active repair is realized through the utilization of AUV assistance and acoustic-optical switching, whereby the negative impacts brought by mobility are effectively offset.

It is noteworthy that a distinct cross point is observed between the curves of PHVP and DROR at a speed of approximately 3.1 m/s. In low-speed scenarios, the hierarchical structure established by PHVP is relatively stable, and more determinate recovery paths can be provided compared to DROR. However, as the speed exceeds 3 m/s, a maintenance lag in the hierarchical structure of PHVP is caused by high-frequency topological changes, leading to a sharp decline in recovery performance. In contrast, a depth-based opportunistic forwarding strategy is adopted by DROR. A higher tolerance for topological deformation is possessed by this structure-free loose routing method; therefore, PHVP is surpassed by DROR in high-speed mobility scenarios. The performance of T-SAPR is slightly inferior to that of DROR. This is primarily because a lag exists in its trust value updates under high-speed mobility, potentially causing nodes that are physically connected but whose trust values have not yet converged to be excluded from repair paths. Furthermore, as static backtracking or local scanning is relied upon by ERR-UWSN and SOVHAR, the “path drift” caused by rapid movement is difficult to adapt to, resulting in the most severe performance attenuation in high-speed scenarios. In the high-dynamic limit scenario with a node mobility speed of 6 m/s, significant robustness is exhibited by HAO-AVP. Compared with DROR, PHVP, T-SAPR, ERR-UWSN, and SOVHAR, the void recovery rate is improved by approximately 18.8%, 22.0%, 27.1%, 40.8%, and 52.5%, respectively.

#### 5.2.3. Analysis of Load Balancing Performance

**Figure 15:**
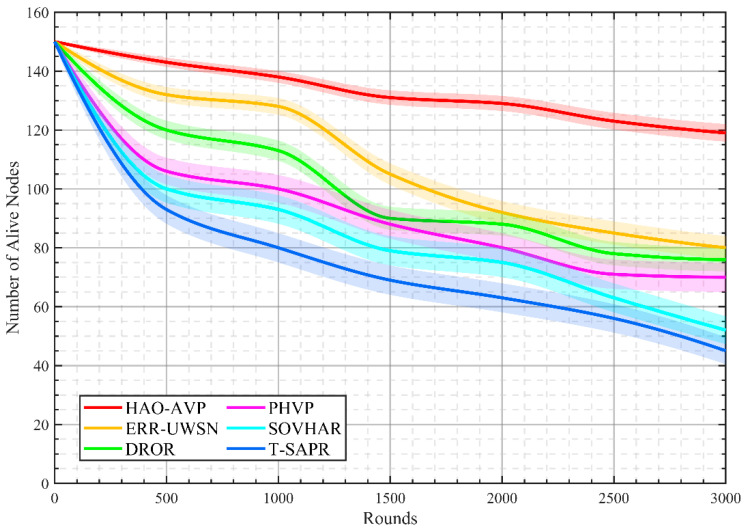
Comparison of the Number of Alive Nodes vs. Simulation Rounds (Number of Nodes = 150).

Figure 15 illustrates the comparison of the number of living nodes as it varies with simulation rounds. Simultaneously, the average trend of the number of living nodes over time (center line) and performance stability (shadow band width) for each protocol are displayed in Figure 15. A relatively gentle decline is shown by the mean curve of the HAO-AVP protocol, and the highest number of living nodes is maintained. More importantly, a narrower shadow band is observed for HAO-AVP compared to all other algorithms, reflecting its higher operational stability. This unification of high performance and high stability is derived from its load balancing mechanism based on Information Entropy and the Gini Coefficient, by which network energy consumption is evenly distributed, thereby significantly extending the network lifetime. In contrast, faster declines in curves and wider shadow bands are exhibited by all comparative protocols, indicating higher performance volatility. Among them, although a certain degree of energy awareness is possessed by ERR-UWSN and DROR, stability is still considered insufficient due to the lack of a global balancing objective. Since no load balancing mechanism is built into PHVP, SOVHAR, and T-SAPR, nodes are prone to randomly becoming hotspots. Consequently, not only is their mean performance relatively lower, but the weakest stability is also demonstrated by their broad band regions. For instance, at the 2000th round, the numbers of living nodes for HAO-AVP, T-SAPR, SOVHAR, PHVP, DROR, and ERR-UWSN are observed to be 129, 63, 75, 80, 88, and 92, respectively.

#### 5.2.4. Analysis of the Effectiveness of the Graded Repair Mechanism

**Figure 16:**
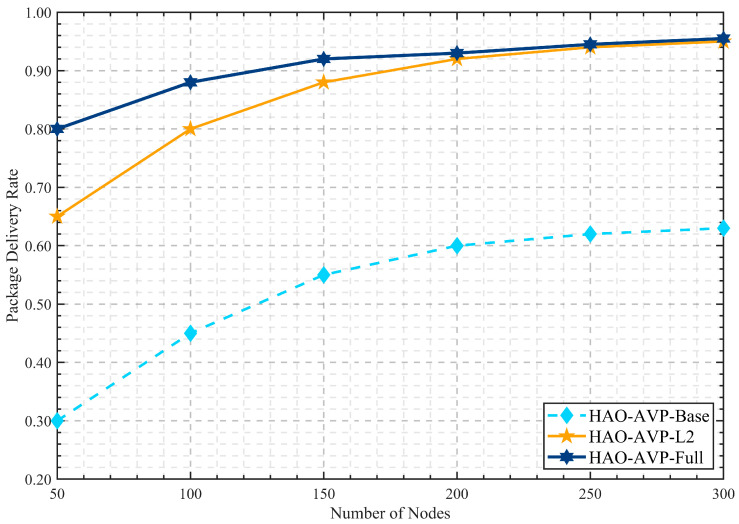
Effectiveness Analysis of the Hierarchical Repair Mechanism.

Figure 16 illustrates the comparison regarding the effectiveness and necessity of the graded repair mechanism. As indicated by the figure, the PDR performance of the protocol configured with three different settings is compared across varying numbers of nodes.

HAO-AVP-Base is comprised solely of reinforcement learning routing, with no repair mechanism included; HAO-AVP-L2 is augmented from the Base version by adding two levels of local repair—optical path adjustment and acoustic-optical switching; whereas HAO-AVP-Full is constituted by a four-level repair mechanism, encompassing optical path adjustment, acoustic-optical switching, path backtracking, and AUV assistance. As illustrated in the figure, the lowest PDR level is exhibited by HAO-AVP-Base across all densities. Particularly in sparse networks (50–100 nodes), a PDR lower than 0.45 is observed, representing suboptimal performance. This indicates that routing voids are identified as a critical bottleneck constraining network reliability; therefore, the necessity of repair mechanisms is demonstrated. Upon the activation of local repair in HAO-AVP-L2, the PDR is increased from 0.45 to 0.80 at 100 nodes, whereby the high efficiency of the optical path adjustment and acoustic-optical switching mechanisms is verified.

It is noteworthy that a PDR superior to that of HAO-AVP-L2 and HAO-AVP-Base is exhibited by the proposed HAO-AVP-Full protocol across all node quantities. The advantage of the HAO-AVP-Full protocol is observed to be more pronounced in sparse networks (50–100 nodes). In such networks, topological voids are found to be more severe, and the local repair capabilities of optical path adjustment and acoustic-optical switching are found to have reached their upper limits. At this juncture, “hard voids” that cannot be resolved by HAO-AVP-L2 are effectively overcome by HAO-AVP-Full by virtue of its unique path backtracking and AUV assistance repair means. Consequently, a PDR of 0.80 is attained at 50 nodes, which is higher than the 0.65 achieved by HAO-AVP-L2. With an increase in the number of nodes (>200 nodes), the curves of HAO-AVP-L2 and HAO-AVP-Full are observed to converge. This is attributed to the fact that in dense networks, the problem is sufficiently addressed by optical path adjustment and acoustic-optical switching. Consequently, the high-cost path backtracking and AUV assistance mechanisms are adaptively reserved, whereby unnecessary resource overhead is avoided. In summary, high reliability and robustness of the protocol under various network densities are ensured by the four-level progressive mechanism of HAO-AVP-Full. It is further indicated that in practical deployments, the most appropriate repair strategy can be selectively configured by balancing specific requirements for PDR reliability against acceptable network overheads (such as the operational cost of AUVs).

#### 5.2.5. Sensitivity Analysis of Reward Function Weights

**Figure 17:**
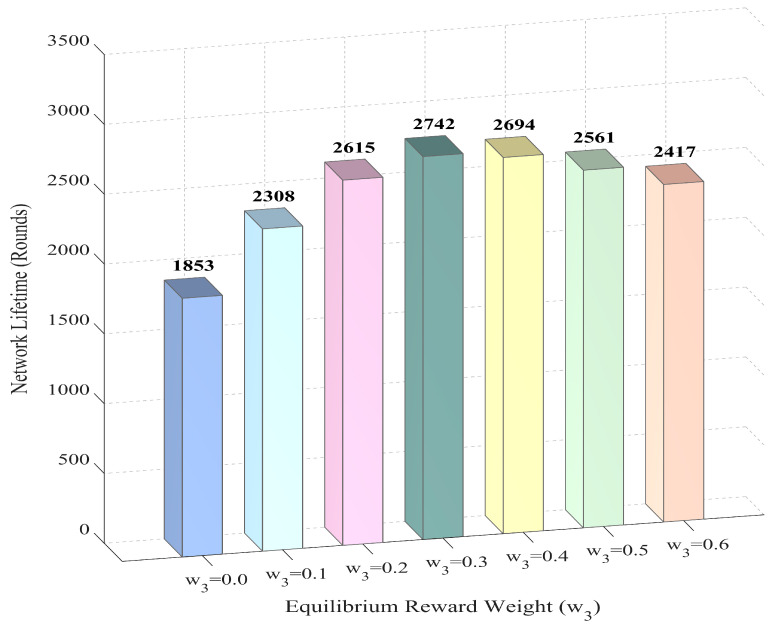
Variation of Network Lifetime with Equilibrium Reward Weight $w_{3}$.

The impact of the weight parameter $w_{3}$ on network performance, specifically the influence of the equilibrium reward term $R_{balance}$ on the network lifetime, is illustrated in Figure 17. To simplify the analysis, the weights are constrained by $w_{1}=w_{2}=(1-w_{3})/2$, and $w_{3}$ is systematically varied from 0 to 0.6 to observe variations in network lifetime. As $w_{3}$ increases from 0, a significant extension in network lifetime is initially observed, reaching a peak within the interval of $w_{3}=0.3∼0.4$. However, when $w_{3}$ is set excessively high (>0.4), a decline in network lifetime is observed.

This non-monotonic trend of network lifetime versus weight provides empirical verification for the theoretical analysis regarding reshaping convergence behavior via the reward function. The regularization strength of $R_{balance}$ within the Q-value landscape is essentially controlled by the weight $w_{3}$. The experimental results clearly demonstrate three distinct stages of convergence behavior:

1. Under-regularization Stage ($w_{3}<0.3$):

When the balance weight is low (or $w_{3}=0$), the regularization constraint is insufficient. The algorithm is primarily driven by the progress reward $R_{prog}$, exhibiting strong greedy characteristics. Agents tend to converge to a few nodes on the geometric shortest path (i.e., falling into local optima), leading to the premature emergence of energy hotspots. This validates the theoretical inference that the absence of $R_{balance}$ leads to winner-takes-all convergence.

2. Optimal Balance Point ($w_{3}\approx 0.3$):

At this point, the optimal trade-off between routing efficiency (gradient depth) and load fairness (gradient breadth) is achieved. The Q-value peaks are moderately smoothed by the regularization term provided by $R_{balance}$, extending the convergence target from a single optimal path to a routing set containing multiple healthy neighbors, thus realizing the maximization of global load balance and network lifetime.

3. Over-regularization Stage ($w_{3}>0.3$):

As $w_{3}$ increases further, a decline in network lifetime is observed. This is attributed to Gradient Dilution caused by excessive regularization. The directional objective is overwhelmed by the balance objective, causing agents to select inefficient paths with excessive hop counts and long physical distances in pursuit of extreme load fairness. The benefits of load balancing are negated by the additional transmission energy consumption. This confirms that excessive $R_{balance}$ weakens the directional guidance of $R_{prog}$.

In summary, the experimental data not only determines the optimal parameter configuration ($w_{3}=0.3$) but also confirms the theoretical mechanism of the Entropy-Gini term acting as a Q-value regularizer from an empirical perspective.

#### 5.2.6. Analysis of Computational Complexity and Engineering Feasibility

To validate the practical deployability of the proposed HAO-AVP protocol on resource-constrained underwater sensor nodes, quantitative evaluations of its computational complexity and memory footprint were conducted in this section. Additionally, lightweight strategies oriented towards engineering implementation were proposed.

1. Complexity Analysis and Resource Consumption Evaluation:

To evaluate the computational overhead of the proposed algorithm, performance tests were performed on the core functional modules using a simulation platform (Intel Core Ultra9, 32 GB RAM). To mitigate stochastic errors associated with single runs, the average code execution time and peak memory usage from 10 independent runs were recorded, and the results are presented in Table 5. As indicated by the data, the steady-state operation components of the protocol (RL routing and Markov prediction) are extremely lightweight; a single decision requires approximately 0.153 ms, with memory usage maintained below 7 KB. Even for the relatively computationally intensive PSO repair module, the execution time was controlled at approximately 45 ms, and the memory usage did not exceed 20 KB. These results demonstrate that the algorithm possesses high execution efficiency on general-purpose computing platforms, providing a solid foundation for subsequent porting to resource-constrained embedded nodes.

2. Deployment-Oriented Lightweight Strategies:

Although the aforementioned evaluations indicate that the baseline overhead of the protocol falls within an acceptable range, considering the severely constrained computational resources and battery capacities of underwater nodes (e.g., the STM32L4 series or MSP430), three specific engineering lightweight strategies are proposed in this paper to further reduce power consumption and optimize real-time performance:

Markov State Space Compression:

The computational complexity of matrix multiplication in the Markov prediction module is proportional to the square of the number of energy states. Although fine-grained discretization levels (S = 100) were utilized in the theoretical model, a coarse-grained scheme (S = 8) was implemented for practical deployment. From a mathematical perspective, floating-point operations are reduced by over 99% through this state reduction. Consequently, sufficient sensitivity for void warning is maintained while CPU cycles are significantly conserved.

Asynchronous Q-Value Update:

In traditional Q-Learning, a Q-value update is executed for every forwarded data packet, leading to frequent write operations on Flash memory. Therefore, an event-triggered asynchronous update mechanism is adopted: a Q-value iteration is triggered only when the magnitude of change in the cumulative reward exceeds a preset threshold or after a fixed time window has elapsed. In high-traffic scenarios, memory I/O frequency and the associated CPU overhead are expected to be reduced by approximately 40% via this strategy, thereby extending the hardware lifespan.

Micro-PSO Implementation:

A Micro-PSO variant is employed for the computationally intensive Level-4 repair. By reducing the particle population size from the standard 50 to 10 and limiting the maximum number of iterations to within 20, the algorithm is constrained to output a suboptimal but feasible solution within a strict time budget. Through this trade-off, it is ensured that the algorithm converges to a feasible suboptimal solution within the strict real-time budget of embedded systems, preventing the triggering of a system watchdog timer timeout.

#### 5.2.7. Multi-AUV Collaboration and Cost–Benefit Sensitivity Analysis

To address the practical constraints of underwater deployment-specifically the trade-offs between repair latency, energy consumption, and hardware costs-this paper investigates the performance of the HAO-AVP protocol under varying AUV densities. A task allocation model is also proposed to resolve scheduling conflicts, alongside an analysis of the cost–benefit ratio (CBR) to determine the optimal triggering threshold.

1. Multi-AUV Task Allocation Model:

When multiple AUVs ($N_{auv}>1$) are available, a centralized bidding strategy is employed to prevent scheduling conflicts. The triggering of an AUV repair task is determined by the Void Severity Score ($S_{k}$), which was previously defined in Equation (40) to quantify the urgency of the routing void based on local sparsity and traffic load. We introduce a variable Trigger Threshold (τ). A physical repair request is broadcast only when the severity score satisfies $S_{k}>\tau$.

Upon receiving a request, the central controller (e.g., the Sink) evaluates the assignment cost $C_{ij}$ for each available AUV a to target void void1:

(52) $$C_{a,void1}=\omega_{t}\cdot \frac{d_{a,void1}}{v_{auv}}+\omega_{e}\cdot \left(1-\frac{E_{rem}^{a}}{E_{init}}\right)$$

where $d_{a,void1}$ is the Euclidean distance, $v_{auv}$ represents the moving velocity of the AUV, $E_{rem}^{a}$ and $E_{init}$ represent the residual energy of AUV a and its initial energy capacity, respectively. $\omega_{t}$ and $\omega_{e}$ are weighting coefficients balancing the travel time and energy cost, satisfying $\omega_{t}+\omega_{e}=1$.

The task is assigned to the AUV with the minimum $C_{ij}$, ensuring that resources are dispatched based on both proximity and energy capabilities.

2. Sensitivity Analysis and Cost–Benefit Trade-offs:

This paper presents a sensitivity analysis by varying the AUV number ($N_{auv}$) and the trigger threshold (τ). The Cost–Benefit Ratio (CBR) is defined as the percentage improvement in Packet Delivery Ratio (PDR) per unit of energy consumed (kJ). Table 6 presents the simulation results. The data reveals a clear trade-off between network reliability and operational cost:

3. Discussion on Engineering Feasibility:

Fallback Scheme (AUV-Absent Scenario):

As shown in the first row of Table 6, when $N_{auv}=0$, the protocol automatically falls back to the Level 3 mechanism (Path Backtracking). Although PDR drops to 82.5%, the network maintains basic connectivity without incurring mechanical movement costs, proving that the protocol is not solely dependent on AUVs.

Optimal Configuration:

The CBR peaks at $N_{auv}=1$ or 2 with a high threshold (τ≥0.6). This indicates that deploying a small number of AUVs to fix only critical “hard voids” yields the maximum return on investment.

Diminishing Returns:

With $N_{auv}=3$ and a low threshold (τ=0.4), the PDR improvement is marginal compared to the surge in energy consumption. This verifies that an aggressive repair strategy is economically inefficient.

#### 5.2.8. Sensitivity Analysis of Optical Link Robustness Under Jerlov Water Types

To validate the adaptability of the optical communication model in realistic underwater environments, the performance of the proposed in-medium adjustment (Level 2 repair) strategy is evaluated under varying water turbidity conditions.

1. Optical Attenuation Model:

The modeling of the optical attenuation coefficient, c(λ), adopts the classic Jerlov water type classification standard. According to the received optical power model defined previously (Equation (11)), the received power exhibits an exponential decay relationship with the attenuation coefficient. Specifically, the value range of c(λ) is set from 0.056 m^−1^ (Type I, clear ocean) to 0.55 m^−1^ (Coastal, turbid). A repair attempt is deemed successful only when the adjusted beam SNR exceeds the decoding threshold ($\Gamma_{th}$).

2. Impact of Turbidity on Repair Success Rate:

The optical repair success rates under different Jerlov water types are presented in Table 7.

Clear Water (Type I-IB): In deep ocean environments ($c(\lambda )<0.1\;m^{-1}$), a success rate exceeding 92% is maintained, indicating the effectiveness of the beam steering mechanism under standard operating conditions.

Transition Zone (Type II): As turbidity increases ($c(\lambda )\approx 0.16\;m^{-1}$), a decrease in the success rate to 78.5% is observed, attributed to the limited effective range caused by scattering.

Turbid Water (Type III-Coastal): In coastal environments ($c(\lambda )>0.39\;m^{-1}$), the optical repair success rate declines to below 45% due to signal attenuation and bio-occlusion effects.

3. Effectiveness of the Hybrid Channel Switching Mechanism:

As indicated in Table 7, the transition from deep ocean (Type I) to turbid coastal environments (Coastal C1) corresponds to an increase in the optical attenuation coefficient, resulting in a reduction in the Level 2 optical repair success rate from 98.5% to 12.6%. However, network disconnection is avoided. The data demonstrates that as the optical link failure rate increases, a complementary rise in the Acoustic Fallback Rate occurs. These results verify that the Hybrid Channel Switching Mechanism adaptively toggles between the high-bandwidth optical mode and the “robust acoustic mode” based on environmental turbidity, thereby ensuring network connectivity is preserved under varying turbidity conditions.

### 5.3. Discussion

#### 5.3.1. Advantages of the Proposed Method

The Entropy-Gini reinforcement learning decision-making is combined with the prediction-identification-repair collaborative mechanism by the HAO-AVP protocol proposed in this paper. Energy efficiency balance in routing, robustness in void handling, and high dynamic adaptability are simultaneously taken into account, making the protocol suitable for complex underwater hybrid acoustic-optical network scenarios.

1. Advantages in Void Identification and Repair: Void boundaries are collaboratively locked by Markov trend prediction and on-demand hop discovery. Means such as optical adjustment, acoustic hopping, and AUV assistance are adaptively matched by the four-level progressive repair strategy. Consequently, the detection of concealed voids, dead-end regions, and large-scale fractures is rendered more accurate, and link recovery is made more stable. Furthermore, under extreme conditions of node sparsity (50 nodes) and drastic topological changes (6 m/s), missed detections and false alarms are effectively suppressed. Identification and recovery rates above 94% and 90%, respectively, are consistently maintained under high dynamics. Stability significantly superior to single-mechanism protocols (such as PHVP and ERR-UWSN) is exhibited when migrating across scenarios (different densities and speeds).
2. Advantages in Energy Efficiency Balance and Latency: Information Entropy and the Gini Coefficient are introduced into the routing decision layer to replace the simple residual energy greedy strategy, whereby the premature death of “hotspot” nodes is reduced. The high bandwidth and low latency characteristics of optical communication are prioritized in the transmission layer, and end-to-end latency is significantly compressed while connectivity is ensured. It is indicated by experiments that the lowest energy consumption Gini coefficient is achieved by HAO-AVP, effectively preventing the generation of energy holes and adapting to long-cycle monitoring and latency-sensitive tasks.
3. Advantages in Dynamic Adaptability and Robustness: A full-process strategy ranging from active source avoidance to mid-stage collaborative prediction to terminal graded repair is adopted. Acute perception of topological evolution is retained, link reconstruction speed is accelerated, and routing oscillation is reduced, facilitating the maintenance of continuous communication in environments with ocean current interference and node drift. By combining active risk warning with multi-modal switching, communication is rendered more stable, and lower packet loss rates and circuit break risks are observed when migrating from low-speed static waters to high-speed dynamic current environments.

#### 5.3.2. Limitations and Future Work

Although stable benefits have been achieved by HAO-AVP in variable simulation environments, algorithm uncertainty may still be amplified by turbidity, complex noise, and micro-node computing constraints in real underwater environments. In particular, a further trade-off and optimization between communication reliability under extremely low SNR and resource consumption for end-side deployment are still required.

1. Challenges in Extreme Underwater Channels: Optical communication distances are severely weakened by strong turbulence, high turbidity, and background optical noise. Link stability is degraded under strict alignment requirements, and the quality of acoustic-optical switching remains to be improved. It is suggested that real water optical characteristic models and adaptive beam divergence control be introduced. Samples containing turbidity and multipath interference should be added during the training phase, and dynamic channel quality thresholds should be adopted during the inference phase to stabilize output.
2. Strong Dependence on Node Computing Power: Heavy calculation loads are incurred by Reinforcement Learning and AUV path planning (PSO). Calculation latency is amplified by long-tail state spaces and frequent Q-value updates, while the storage and energy consumption of micro-sensor nodes are limited. It is suggested that “cloud-edge-end” collaborative computing be implemented. Complex training tasks should be transferred to sink nodes or surface buoys, and lightweight RL models and operator pruning should be utilized to alleviate the computational burden on single nodes.
3. Optimization of AUV Scheduling Costs: Although a high repair rate is yielded by AUVs, significant navigation energy consumption and scheduling time costs are incurred. All breakpoints are difficult to cover by a single AUV in ultra-large-scale networks. It is suggested that research on multi-AUV collaboration and task allocation algorithms be conducted. Energy harvesting technologies (such as thermal energy and wave energy) should be combined to reduce the comprehensive cost of physical repair without sacrificing the success rate of recovery.

To address the aforementioned issues, lightweight neural networks and transfer learning will be introduced in subsequent work to reduce reliance on computing power. The realism of channel models will be enhanced by combining tank experiments with lake trial data. Distributed multi-agent collaboration will be conducted to reduce latency while maintaining accuracy, and anti-interference coding for underwater acoustic communication will be explored to reinforce connectivity rates under weak signal conditions.

#### 5.3.3. Scenario Expansion

Good transferability is possessed by the multi-modal collaborative and adaptive repair mechanisms of HAO-AVP, allowing them to be reused in various underwater infrastructure scenarios characterized by “high dynamics, limited energy, and harsh communication environments.” Through parameter fine-tuning and minor strategy adaptations, stable transmission can be maintained under different operating conditions, providing reliable link support for the closed-loop of the subsequent Marine Internet of Things.

1. Smart Marine Ranching Monitoring: Frequent blocking of optical paths is caused by high breeding density and fish shoal movement. High-definition video and environmental parameters can be stably transmitted back by adopting rapid switching with optical priority and acoustic backup. Combined with energy efficiency balance strategies, node failure and maintenance frequency under continuous monitoring are synchronously compressed, making it suitable for multi-source networking of fixed pile foundations and mobile inspection torpedoes.
2. Underwater Military Defense and Tactical Reconnaissance: High concealment is required in highly antagonistic battlefield environments. Load concealment is reinforced by the Entropy-Gini reward function, and the probability of detection by sonar is reduced by the silent characteristic of optical communication. Combined with active void avoidance, long survival cycles are balanced with high-reliability intelligence transmission. When combined with underwater vehicle formations, a closed-loop command and control system of anomaly detection-silent transmission-collaborative strike can be formed.
3. Deep-sea Oil/Gas Pipeline and Cable Inspection: The target area is narrow and long with a large depth span. Physical voids caused by cable breaks can be precisely handled by AUV assistance in the four-level repair strategy. Combined with path backtracking and multi-hop relaying, long-distance inspection data relay can be realized in deep-sea areas without infrastructure. Furthermore, routing interruption risks can be directly mapped to maintenance work orders and emergency repair priority sequencing through early warning mechanisms.

## 6. Conclusions

This paper proposes the HAO-AVP protocol to address routing voids and energy constraints in UWSNs. The Gini Coefficient and Information Entropy are integrated into the Reinforcement Learning reward function to achieve energy fairness and load balancing. Additionally, a prediction–identification–repair mechanism is designed, employing a four-level strategy (optical adjustment, acoustic-optical switching, backtracking, and AUV assistance) to handle routing voids. Simulation results validate the protocol’s effectiveness in complex underwater environments. Experimental data indicates that in high-density scenarios with 350 nodes, extremely high reliability is exhibited by HAO-AVP. Compared with PHVP, DROR, T-SAPR, ERR-UWSN, and SOVHAR, the void identification rate is improved by approximately 7.6%, 8.4%, 13.8%, 19.5%, and 25.3%, respectively, and the void recovery rate is improved by approximately 4.3%, 9.6%, 12.0%, 18.4%, and 24.2%, respectively. In addition, existing mainstream protocols are also outperformed by HAO-AVP in key metrics such as network lifetime, average end-to-end delay, and load balancing. Although certain results have been achieved in this paper, several directions remain to be explored in depth in future work. First, efforts will be dedicated to constructing a more refined dynamic acoustic-optical channel model, where physical environmental factors such as water turbulence and suspended particle turbidity are incorporated into the routing decision considerations. Second, to address the computational challenges of large-scale heterogeneous networks, Deep Reinforcement Learning (DRL) or Distributed Multi-Agent Reinforcement Learning (MARL) algorithms are planned to be introduced to enhance algorithm scalability and convergence speed. Finally, considering the security requirements of underwater environments, the integration of trust evaluation and anti-attack strategies into the HAO-AVP framework to construct a comprehensive routing system with high reliability, strong robustness, and security is also identified as an important direction for subsequent research.

## Figures and Tables

**Table 1:** Comparison of characteristic dimensions between TWSN and UWSN.

Characteristic Dimension	TWSN	UWSN
Communication Medium	RF	Acoustic waves, Underwater optical waves
Propagation Speed	Extremely high (~3 × 10^8^ m/s)	Extremely low (~1.5 × 10^3^ m/s, Acoustic)
Propagation Delay	Extremely low, negligible	Extremely high;
Available Bandwidth	Wide (MHz~GHz)	Extremely narrow (kHz level, Acoustic)
Network Topology	Mostly 2D, relatively static	3D, highly dynamic due to water currents
Node Localization	GPS available	GPS unavailable
Node Cost & Density	Low cost; dense deployment achievable	High cost; typically sparse deployment
Channel Quality	Relatively reliable	severely affected by multipath effects and noise
Void Characteristics	Mostly static; caused by obstacles or initial deployment	Dynamic and mobile; caused by node energy depletion and movement
Applicability of Void Handling Schemes	Based on 2D geometry; relying on static path discovery (e.g., planar graph traversal)	Requires 3D spatial awareness; adaptable to dynamic topology

**Table 2:** List of Symbols and Notations.

Symbol	Description	Symbol	Description
Network & Channel Model (Equations (1)–(19))			
$d_{ij}$	Transmission distance	*λ*	Optical wavelength
f	Frequency of acoustic wave	c(λ)	Beam attenuation coefficient
$\tilde{k}$	Spreading factor, typically 1.5 or 2	R	Responsivity
α(f)	Absorption coefficient	$\sigma_{shot}^{2}$	Shot noise variance
SL	Source Level	$\sigma_{thermal}^{2}$	Thermal noise variance
NL	Noise Level	q	Elementary charge
DI	Directivity Index	B	Bandwidth
$N_{t}(f),\;N_{s}(f)$	Turbulence/Shipping noise	$I_{bg}$	Background light irradiance
$N_{w}(f),\;N_{th}(f)$	Wave/Thermal noise	$I_{d}$	Detector dark current
s	Shipping activity factor	$k_{B}$	Boltzmann constant
w	Wind speed	T	Absolute temperature
$P_{tx\_a}$ $P_{rx\_a}$	Acoustic transmission power Acoustic reception power consumption	$E_{tx\_a}\left(L,d_{ij}\right)$ $E_{rx\_a}(L)$	Energy consumption for underwater acoustic comm. transmission/reception
$SNR_{th}$	Min SNR threshold	$R_{L}$	Load resistance
$P_{elec}$	the fixed power consumption for circuit processing	$R_{a}$ $R_{o}$	acoustic comm. rate optical comm. rate
$P_{rx_{\_opt}}$ $P_{tx\_opt}$	Received optical power Optical transmission power	$E_{tx\_o}\left(L,d_{ij}\right)$ $E_{rx\_o}(L)$	Energy consumption for underwater optical comm. transmission/reception
$\eta_{t},\eta_{r}$	Optical efficiencies	$E_{init}$	initial energy
$A_{rx}$	Receiver aperture area	$E_{rem}\left(v_{i},t\right)$	residual energy
*θ*	Beam divergence angle	$v_{i}$	node
$A_{beam}(d_{ij})$	Beam area at distance d	${N(v}_{i})$	neighbor nodes of $v_{i}$
$SNR_{opt}$	the SNR at the receiver	${d(v}_{i},v_{s})$	distance of $v_{i}$ and $v_{j}$
Proposed HAO-AVP Protocol (Equations (20)–(52))			
$s_{i}$	$\mathrm{State}\;\mathrm{vector}\;\mathrm{of}\;v_{i}$	η	the risk aversion coefficient
$E_{rem}(v_{i})$	Normalized residual energy	σ	Penalty Range Parameter
${D(v}_{i})$	$\mathrm{Euclidean}\;\mathrm{distance}\;\mathrm{from}\;v_{i}$ to sink	$\lambda_{1}$	Penalty Intensity
$A(s_{i})$	$\mathrm{the}\;\mathrm{action}\;\mathrm{space}\;\mathrm{of}\;v_{i}$	$w_{sev}$	Weight for Severity
$N(v_{i})$	$\mathrm{neighbor}\;\mathrm{nodes}\;\mathrm{of}\;v_{i}$	$w_{aff}$	Weight for Affected Scope
$Q_{len}(v_{i})$	the packet queue length	$w_{qlen}$	Weight for Queue Length
$Q_{t}(s_{i},a_{j})$	Q-value function	$w_{diet}$	Weight for Distance
α	Learning rate	$w_{devv}$	Weight for Deviation
$R(s_{i},a_{j})$	the immediate reward	$Count_{bt}(k)$	Backtrack Counter
γ	Discount factor	$\left|N_{aff}(k)\right|$	Number of Affected Neighbors
$\underset{a^{\prime }\in A(s_{j})}{max}\;Q_{t}(s_{j},a^{\prime })$	the maximum potential future Q-value	${\bar{Q}}_{len}(k)$	Average Queue Length
$A(s_{j})$	$\mathrm{the}\;\mathrm{action}\;\mathrm{space}\;\mathrm{of}\;\mathrm{node}\;\mathrm{of}\;v_{j}$	$d(P_{AUV},Loc_{k})$	Navigation Distance
$w_{1},w_{2},w_{3}$	Weighting coefficients	$D_{max}$	Max Navigation Range
$R_{prog}(v_{j})$	$\mathrm{Progress}\;\mathrm{Reward}\;\mathrm{of}\;v_{j}$	$E_{travel}(k)$	Travel Energy Consumption
$R_{energy}(v_{j})$	$\mathrm{Energy}\;\mathrm{Reward}\;\mathrm{of}\;v_{j}$	$E_{AUV\_rem}$	Remaining Energy of AUV
$R_{balance}({v_{i},v}_{j})$	Equilibrium Reward of $v_{i}$ and $v_{j}$	${\bar{Q}}_{len}(k)$	Average Queue Length
$w_{b}(v_{i})$	Dynamic Weighting Factor	Dev(k)	Deviation Degree
$H_{energy}(v_{i})$	Energy Information Entropy	F(P)	Fitness Function
k	adjustment parameter	P	Candidate Position
$\sigma_{E}(v_{i})$	standard deviation of the residual energy	$M_{conn}(P)$	Metric of Connectivity
$G_{load}(v_{i})$	Forwarding Load Gini Coefficient	$w_{c1}$	Weight for Connectivity
$p_{k}$	The energy proportion	$w_{d}$	Weight for Distance
${\bar{E}}_{rem}$	Average residual energy	$P_{AUV}$	Current Position of AUV
$L_{k}$	the number of data packets forwarded	$\Theta_{m}(t)$	Particle Velocity
∑	Summation symbol	$\tilde{\omega }$	Inertia Weight
$\bar{L}(v_{i})$	the average load	$c_{1},c_{2}$	Learning Factors
$\tilde{q}$	Random Number	$r_{1},r_{2}$	Random Numbers
ε	Epsilon/Exploration Rate	$P_{best,m}$	Personal Best Position
$\pi_{j}^{\left(k\right)}$	$\mathrm{The}\;\mathrm{state}\;\mathrm{probability}\;\mathrm{vector}\;\mathrm{of}\;\mathrm{node}\;n_{j}$ after k time steps.	$P_{m}(t)$	Current Position
$\pi_{j}^{\left(0\right)}$	$\mathrm{the}\;\mathrm{initial}\;\mathrm{state}\;\mathrm{probability}\;\mathrm{distribution}\;\mathrm{vector}\;\mathrm{of}\;n_{j}$	$G_{best}$	Global Best Position
$(P_{j}{)}^{k}$	$\mathrm{The}\;\mathrm{one-step}\;\mathrm{state}\;\mathrm{transition}\;\mathrm{probability}\;\mathrm{matrix}\;\mathrm{of}\;n_{j}$	$P_{m}(t+1)$	Next Position
k	Predicted time step	$C_{relay}$	Cost of Relay Mode
$S_{crit}$	Critical/Endangered State	$C_{deploy}$	Cost of Deployment Mode
$P_{void}$	Joint Probability	$P^{*}$	Optimal Position
$P_{th}$	Void Warning Threshold	${FP(v}_{j})$	$\mathrm{Forward}\;\mathrm{potential}\;\mathrm{of}\;v_{j}$
$N_{prog}^{\prime }$	Set of Uncovered Progress Neighbors	$N_{a}(v_{i})$	$\mathrm{The}\;\mathrm{acoustic}\;\mathrm{neighbors}\;\mathrm{of}\;v_{i}$
$N_{o}(v_{i})$	Optical Neighbor Set	$P_{b}$	the Bit Error Rate (BER)
$N_{covered}$	Set of Covered Neighbors	$A^{\prime }(s_{p})$	New Action Space
$\theta_{new}$	The minimum required new	ϵ	Minimal constant
$\phi_{ij}$	the deviation angle	$s_{p}$	State of Previous Hop
$\delta_{margin}$	the angular margin	$LS(v_{p},v_{k})$	Link Stability
$C_{1}(\theta_{new})$	The energy cost	$C_{travel}(P^{*})$	Travel Cost
$\theta_{old}$	Original Divergence Angle	$E_{rem\_avg}(v_{j})$	$\mathrm{Average}\;\mathrm{Residual}\;\mathrm{Energy}\;\mathrm{of}\;v_{j}$
$\theta_{old}$	Original Divergence Angle	PAPR $\left(d(v_{i},v_{j}),f\right)$	Predicted Acoustic Packet Reception Rate
$w_{q}$	Potential Energy Term Weighting	$C_{op}$	Operational Cost
$w_{r}$	Reliability Weighting	$T_{stay}$	Stay Duration
$w_{c}$	Cost Weighting	$C_{node}$	Node Cost
CDUE	Utility value/score	Action	Decision Result

**Table 3:** Software and Hardware Environment Configurations for Simulation Experiments.

Name	Setting
CPU	Intel(R) Core(TM) Ultra9-185H
Frequency	2.30 GHz
RAM	32.0 GB
Hard drive	1 TB
GPU	NVIDIA GeForce RTX 4060 Laptop GPU
VRAM	8.0 GB
Operating system	Windows 11
Language	MATLAB R2019b

**Table 4:** Simulation Parameters.

Parameter Category	Parameter Name	Value/Description
Network Parameters	Simulation Area	$1000\times \;1000\;\times 1000\;m^{3}$
	Number of Nodes	50–300
	Initial Energy of Nodes	1000 J
	Node Mobility Speed	0–5 m/s
Communication Parameters	$\mathrm{Max}\;\mathrm{Acoustic}\;\mathrm{Range}\;R_{a}$	800 m
	$\mathrm{Max}\;\mathrm{Optical}\;\mathrm{Range}\;R_{o}$	80 m
	Acoustic Data Rate	5 kbps
	Optical Data Rate	100 Mbps
Protocol Parameters	Learning Rate α	0.1
	Discount Factor γ	0.9
	constant ϵ	Initially 1.0, decaying over time
	$\mathrm{Reward}\;\mathrm{Function}\;\mathrm{Weights}\;w_{1}$ $,\;w_{2}$ $,\;w_{3}$	0.4, 0.3, 0.3
	AUV Speed	5 m/s

**Table 5:** Computational Complexity and Memory Footprint of the Algorithm.

Module	PC Runtime (Sim)	Memory (RAM)
RL Routing	0.008 ± 0.002 ms	2.45 KB
Markov Prediction	0.145 ± 0.012 ms	4.12 KB
PSO Repair (Lvl 4)	45.32 ± 2.15 ms	18.6 KB
Routine Total	≈0.153 ms	<10 KB

**Table 6:** Sensitivity Analysis of Multi-AUV Configuration and Cost–Benefit Ratio.

$N_{auv}$	τ	Task Allocation	Total Energy Cost (J)	CBR	PDR
0 (No AUV)	N/A	Fallback Scheme: Purely relying on acoustic backtracking (Level 3).	1520	N/A	82.5%
1 (Single)	High (τ = 0.8)	Greedy: Only triggers repair when void severity ${\;S}_{k}>0.8$ (critical voids).	1850	Optimal	88.4%
2 (Multi)	Medium (τ = 0.6)	Collaborative: Triggers repair for moderate voids (${\;S}_{k}>0.6$); partition-based coverage.	2140	Good	93.1%
3 (Multi)	Low (τ = 0.4)	Aggressive: Triggers repair even for minor voids ($S_{k}>0.4$); high mobility cost.	2780	Diminishing	94.5%

**Table 7:** Impact of Jerlov Water Types on Optical Repair Success and Channel Switching.

Jerlov Water Type	Attenuation Coeff. c(λ)(m^−1^)	Optical Repair Success Rate	Acoustic Fallback Rate	Environment Description
Type I	0.056	98.50%	1.50%	Extremely Clear (Deep Ocean)
Type IA	0.078	96.20%	3.80%	Clear Ocean
Type IB	0.096	92.40%	7.60%	Moderate Ocean
Type II	0.16	78.50%	21.50%	Coastal Waters (Clear)
Type III	0.39	45.20%	54.80%	Coastal Waters (Turbid)
Coastal (C1)	0.55	12.60%	87.40%	Harbor (High Turbidity)

## Data Availability

The original contributions presented in this study are included in the article. Further inquiries can be directed to the corresponding author.
